# The E1B19K-deleted oncolytic adenovirus mutant AdΔ19K sensitizes pancreatic cancer cells to drug-induced DNA-damage by down-regulating Claspin and Mre11

**DOI:** 10.18632/oncotarget.7310

**Published:** 2016-02-10

**Authors:** Constantia Pantelidou, Gioia Cherubini, Nick R. Lemoine, Gunnel Halldén

**Affiliations:** ^1^ Centre for Molecular Oncology, Barts Cancer Institute, Queen Mary University of London, London, UK

**Keywords:** checkpoint-inactivation, apoptosis, mitotic aberrations, pancreatic cancer, cytotoxic drugs

## Abstract

Adenovirus-mediated sensitization of cancer cells to cytotoxic drugs depends on simultaneous interactions of early viral genes with cell death and survival pathways. It is unclear what cellular factors mediate these interactions in the presence of DNA-damaging drugs. We found that adenovirus prevents Chk1-mediated checkpoint activation through inactivation of Mre11 and downregulation of the pChk1 adaptor-protein, Claspin, in cells with high levels of DNA-damage induced by the cytotoxic drugs gemcitabine and irinotecan. The mechanisms for Claspin downregulation involve decreased transcription and increased degradation, further attenuating pChk1-mediated signalling. Live cell imaging demonstrated that low doses of gemcitabine caused multiple mitotic aberrations including multipolar spindles, micro- and multi-nucleation and cytokinesis failure. A mutant virus with the anti-apoptotic E1B19K-gene deleted (AdΔ19K) further enhanced cell killing, Claspin downregulation, and potentiated drug-induced DNA damage and mitotic aberrations. Decreased Claspin expression and inactivation of Mre11 contributed to the enhanced cell killing in combination with DNA-damaging drugs. These results reveal novel mechanisms that are utilised by adenovirus to ensure completion of its life cycle in the presence of cellular DNA damage. Taken together, our findings reveal novel cellular targets that may be exploited when developing improved anti-cancer therapeutics.

## INTRODUCTION

Replication-selective oncolytic adenoviral mutants are promising as future cancer therapeutics because of their efficient lysis of a broad range of adenocarcinoma types, including drug-resistant cancers, with demonstrated clinical safety [1, 2]. Most studies have employed mutants based on adenovirus serotype 5 of species C (Ad5), which has a small 36kb linear dsDNA genome enclosed by a protein capsid [3]. Cellular entry occurs via attachment to Coxsackie virus and adenovirus receptor (CAR), and αvβ3 and αvβ5 integrins for endosome-mediated internalisation and capsid degradation, followed by transport of viral DNA to the nucleus for expression of early viral genes. To support viral DNA and protein synthesis, the early viral E1A gene-products inactivate the G1/S checkpoint, mainly through inhibition of pRb, leading to E2F-mediated transcription of S-phase genes, which is essential for the viral life cycle [4]. To avoid elimination of the infected cells by cellular defence mechanisms, the early viral E1B, E3 and E4 gene-products act to inhibit apoptosis, immune response activation and DNA-damage repair. These viral functions have been exploited to engineer oncolytic mutants that are unable to propagate in normal cells but efficiently replicate in cancer cells with deregulated cell survival and apoptosis pathways [1, 2]. Currently, the clinically most efficacious replication-selective mutants harbour a small deletion of the E1ACR2-domain (e.g. *dl*922-947 and AdΔ24) to ablate pRb-binding that results in potent anti-tumour efficacy in various solid tumours with deregulated cell cycle and limited toxicity to normal tissue (e.g. [5, 6]). To date, significant clinical responses have only been reported in combination with cytotoxic drugs and/or radiation therapy [1, 5, 7-9].

In preclinical models, efficacy of viruses has been clearly demonstrated both when administered alone and in combination with conventional therapeutics. For example, potent synergistic cancer cell killing was demonstrated with several oncolytic adenoviruses in combination with DNA-damaging agents, including gemcitabine and irinotecan [10-13]. However, the cellular mechanisms for the increased tumour cell killing in combination with drugs are mostly unknown, both in preclinical models and in patients. Virus-induced cell lysis occurs through non-apoptotic, necrotic-like cell death mechanisms while in combination with cytotoxic drugs, drug-induced apoptosis is often enhanced [11, 14-16]. It has been established that viral E1A expression is necessary for synergistic enhancement of cytotoxic drug-induced cell death and that E1A-binding to p300 and p400 is required, but not to pRb [14, 17-19]. Furthermore, the E4orf3 and E4orf6 genes are rapidly expressed after infection to prevent the cellular DNA-damage response (DDR) mainly by inactivating the Mre11-Rad50-Nbs1 (MRN) complex [20, 21]. E4orf3 sequesters the Mre11 subunit to nuclear tracks and E4orf6 together with E1B55K targets Mre11, Nbs1 and p53 for proteasomal degradation, resulting in checkpoint abrogation [20, 22, 23]. These functions are likely to contribute to the enhanced cell killing in combination with DNA-damaging drugs.

To take advantage of the virus-mediated enhancement of cancer cell killing in combination with DNA-damaging drugs we developed mutants with the anti-apoptotic E1B19K gene deleted in addition to tumour-selective deletions including the E1ACR2-domain (e.g. AdΔΔ) [11, 24, 25]. E1B19K is a functional Bcl-2 homologue that binds Bax and Bak, therefore inhibiting mitochondrial pore formation and apoptosis in response to both death receptor–induced signaling and intrinsically induced apoptosis (p53-dependent and -independent) [26-29]. Our approach to eliminate E1B19K rather than the anti-apoptotic p53-binding E1B55K (e.g. Onyx-015, H101), which greatly attenuates viral production, resulted in mutants with retained high potency in models of pancreatic and prostate cancers [11, 24]. Both AdΔ19K and AdΔΔ greatly enhanced cell killing induced by the DNA-damaging agents gemcitabine and irinotecan [10, 11], which are currently being used for the treatment of pancreatic cancers.

In the current study, we employed AdΔ19K (deleted only in E1B19K) to identify virus-dependent mechanisms that converge on drug-induced signalling pathways to confer cell death of pancreatic cancer cells. We demonstrate that AdΔ19K cooperates with gemcitabine or irinotecan to deregulate cell-cycle mechanisms. For the first time, we report that adenovirus attenuates Chk1 activation even in the presence of cytotoxic drugs and high levels of DNA damage. We demonstrate that AdΔ19K inactivates the DNA repair factor Mre11 and prevents drug-induced accumulation of Claspin, a protein required for Chk1 activation. These findings identify novel mechanisms for virus-mediated weakening of the DDR, followed by increased mitotic aberrations and cell death, as causes of the virus-mediated drug-sensitization. We conclude that Claspin and associated regulatory factors including Mre11 and Chk1 could be therapeutically targeted by E1B19K-deleted oncolytic viruses and/or novel inhibitors to better manage treatment-insensitive pancreatic cancers in particular.

## RESULTS

### The adenoviral mutant AdΔ19K synergises with gemcitabine and irinotecan by enhancing apoptotic death in pancreatic cancer cells

**Figure 1:**
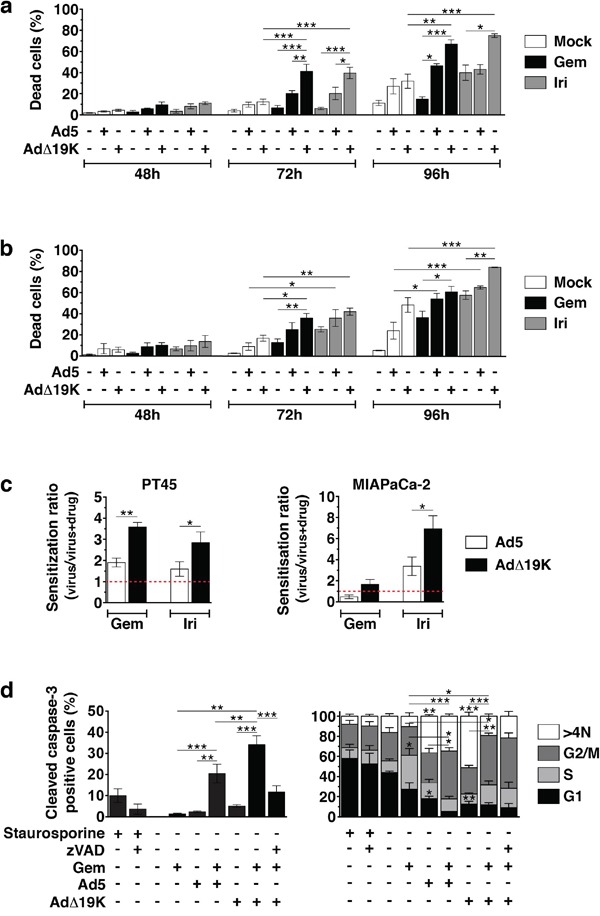
The adenoviral mutant AdΔ19K synergises more potently than Ad5 with gemcitabine and irinotecan by enhancing apoptotic death in pancreatic cancer cells **a** and **b.** Cell death assays using Trypan blue dye incorporation in PT45 (a) and MIAPaCa-2 (b) cells. Error bars represent S.E.M. of A. 3-7 and B. 3-4 independent experiments. **c.** Sensitization ratios (EC_50_ of virus / EC_50_ of virus and drug) derived from cell viability assays in PT45 **(left panel)** and MIAPaCa-2 **(right panel)** cells. Red dotted lines indicate a ratio of 1 (= no sensitization). Drug cytotoxicity (%) ± S.E.M. was: 40.3±5.8% and 30.7±10.7% with 2nM (PT45) and 10nM (MIAPaCa-2) gemcitabine (Gem), respectively, and 28.0±2.1% and 34.3±8.6% with 3μM (MIAPaCa-2) and 5μM (PT45) irinotecan (Iri), respectively. Error bars represent S.E.M. of 4 independent experiments. **d.** Percentage of apoptotic cells (total apoptosis, **left panel**) as determined by cleaved caspase-3 flow-cytometric assays in PT45 cells. Apoptosis in each cell-cycle phase/DNA content **(right panel)** is expressed as % of total apoptosis in each condition. Error bars represent S.E.M. of 3 independent experiments. **a-d.** *.p<0.05, **.p<0.01, ***.p<0.001 (one-way ANOVA with Bonferroni's multiple comparison test).

We previously demonstrated that E1B19K-deleted mutants cause potent synergistic cell death in combination with several cytotoxic drugs including gemcitabine [10, 11]. To investigate the mechanisms of action in the current study we first assessed cell death over time in response to low doses of Ad5 and AdΔ19K in combination with gemcitabine or irinotecan in the pancreatic cancer cells PT45 and MIAPaCa2. In PT45, the combination of AdΔ19K with gemcitabine induced significantly more cell death than each agent alone at 72 through 96h post-treatment (Figure 1a). Similar results were obtained with irinotecan. Cell death with gemcitabine and AdΔ19K was synergistic and higher than the combination with Ad5 at 48 - 96h (Figure 1a and Table S1). In MIAPaCa-2 cells, cell killing was also increased with AdΔ19K in combination with either drug (Figure 1b and Table S1). In accordance, significantly higher sensitization ratios were observed when suboptimal doses of AdΔ19K were combined with gemcitabine or irinotecan compared to Ad5 in cell viability assays, in both PT45 and MIAPaCa2 cells (Figure 1c). We confirmed that the enhanced cell killing occurred through caspase-3-dependent apoptosis that was significantly greater in AdΔ19K-infected gemcitabine-treated cells compared to all other treatments (Figure 1d). Ad5 in combination with gemcitabine also induced caspase-3–dependent apoptosis that was significantly higher than the corresponding single agent-treatments. Interestingly, the majority of apoptosis occurred in cells with a DNA content of 4N and >4N, which mainly represent cells in G2/M phases (Figure 1d). There were significantly more apoptotic cells with 4N DNA content when AdΔ19K was combined with gemcitabine compared to the drug treatment alone. In contrast to AdΔ19K, which caused apoptosis mostly in cells with >4N DNA content, gemcitabine caused apoptosis equally in cells occupying G1, S and G2/M phases and the presence of AdΔ19K promoted the apoptosis of cells in G2/M phases (Figure 1d). Similar trends were observed in Ad5-infected cells.

These findings demonstrate that the synergistic cell killing in AdΔ19K-infected drug-treated cells occurs through more potent caspase-3-dependent apoptosis than in Ad5-infected cells. Furthermore, the enhanced cell killing is independent of viral DNA replication, demonstrated by prevention of both viral DNA amplification and assembly of viral replication centres (Figure S1a-b). In contrast, more adenovirus-infected E1A-positive cells were detected in gemcitabine-treated PT45 and MIAPaCa-2 cells, an increase that occurred earlier in AdΔ19K-infected cells and was paralleled by higher E1A mRNA levels (Figure S1c-d).

### Adenovirus increases the mitotic index and aberrant mitosis in gemcitabine-treated cells

**Figure 2:**
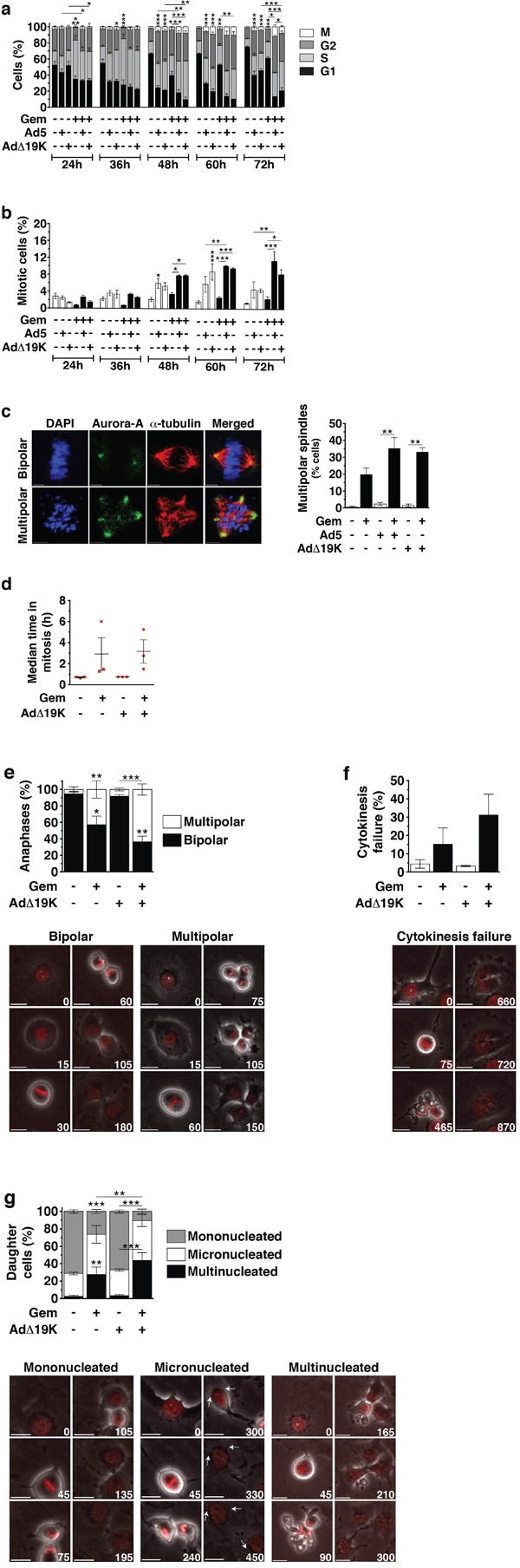
Adenovirus increases the mitotic index in gemcitabine-treated cells and enhances aberrant passage through mitosis **a** and **b.** Cell cycle and mitotic index analysis in synchronised PT45 cells, stained with propidium iodide (for DNA-content analysis), a phospho-histone H3 antibody (for mitotic index analysis) and an E1A antibody (for identification of infected cells) and analysed by flow-cytometry. Dead cells, as identified from their incorporation of FVD, and cells in subG1 phase were excluded from the analysis. **a.** Cell-cycle distribution of non-infected or infected cells, shown as % cells in G1-phase, S-phase, G2-phase and mitosis (M). Only S-phase statistical significance is shown. **b.** Mitotic Index (% cells in mitosis). Error bars represent S.E.M. of 4 independent experiments. **c.** Immunofluorescence microscopy analysis of Aurora-A (green) and α-Tubulin (red) with nuclear DAPI staining (DNA, blue) in PT45 cells fixed at 48hpi using methanol. **Left panel:** Example images of bipolar and multipolar spindles in cells treated with gemcitabine. Scale bar: 5μm. **Right panel:** Quantification of spindle multipolarity, expressed as frequency (%), ≥ 150 mitotic cells/condition were counted. Error bars represent S.E.M of two independent experiments. **d-g.** Time-lapse microscopy 24-96hpi in PT45 cells stably expressing histone H2B-mCherry. Error bars represent S.E.M of 3 independent experiments. Numbers on images indicate time (in minutes). Scale bar: 20μm. **d.** Scatter plot showing the duration of mitosis, defined from the time of nuclear envelope breakdown until the time of sister chromatid separation. At least 100 mitotic cells were analysed/condition/study in 3 independent studies. **e.** Frequency (%) **(top panel)** and example images **(bottom panels)** of bipolar and multipolar anaphases. At least 100 mitotic cells were analysed/condition/study in 3 independent studies. **f.** Frequency (%) **(top panel)** and example images **(bottom panels)** of cytokinesis failure. At least 100 mitotic cells were analysed/condition/study in 3 independent studies. **g.** Frequency (%) (top panel) and example images (bottom panels) of mononucleated, micronucleated and multinucleated daughter cells. At least 150 daughter cells were analysed/condition/study in 3 independent studies. **a-g.** *.p<0.05 **.p<0.01, ***.p<0.001 (one-way ANOVA with Bonferroni's multiple comparison test)

Based on our findings that more apoptotic cells accumulated in G2/M phases we hypothesised that cell cycle progression was required after gemcitabine-induced S-phase arrest for virus to enhance cell killing. To monitor cell cycle progression following treatment, we synchronised cells using thymidine block and performed cell cycle and mitotic index analysis over time, with E1A as a marker of infected cells. As expected, gemcitabine induced S-phase arrest that peaked at 36h; this was followed by cell progression to G2 and a gradual G1 arrest (Figure 2a, statistics in Table S2). AdΔ19K infection did not affect drug-induced S-phase arrest, however, the gradual gemcitabine-dependent G1 arrest (48-72h) was prevented by virus and cells accumulated in S and G2 phases (Figure 2a). In non-synchronised PT45 and MIAPaCa-2 cells gemcitabine-induced S-phase arrest was also unaffected by adenovirus infection and cells accumulated in S and G2/M phases (Figure S2a-b). Similarly, AdΔ19K did not interfere with irinotecan-induced S/G2 arrest (Figure S2c). Interestingly, mitotic index analysis in synchronised PT45 cells revealed that adenovirus infection significantly increased the mitotic index of gemcitabine-treated cells following the drug-induced S-phase block (Figure 2b).

The observation that virus-infection of gemcitabine-treated cells resulted in a higher proportion of cells accumulating in the G2/M phase, prompted us to investigate whether these cells displayed normal or aberrant mitosis. We found that gemcitabine-treated cells exhibited significantly increased spindle multipolarity in mitosis, as determined by immunofluorescence microscopy of the spindle pole marker Aurora A (Figure 2c). Further increases were seen in the presence of either Ad5 or AdΔ19K (Figure 2c), which can be attributed to E1A-induced centrosome amplification [30]. Multipolar spindles were also abundant in irinotecan treated cells (Figure S2d). Since there were no major differences in effect on mitosis and multipolarity between Ad5 or AdΔ19K in combination with gemcitabine we pursued further in depth studies using the AdΔ19K mutant. Analysis of mitotic progression was performed using long-term time-lapse microscopy of synchronised histone H2B mCherry-expressing PT45 cells. Gemcitabine treatment resulted in prolonged mitosis (2.9±1.5h), which was unaffected by AdΔ19K infection (3.2±1.1h) (Figure 2d and S2e). Spindle multipolarity was evident following gemcitabine treatment and resulted in multipolar divisions (42.9±10.6%), with more multipolar anaphases in the presence of AdΔ19K (63.4±6.8%) (Figure 2e). Moreover, gemcitabine treatment induced cytokinesis failure (15.3±8.8%), which was promoted by AdΔ19K (31.1±11.4%) (Figure 2f). As a consequence of the aberrant mitotic progression, a high degree of micro- and multi-nucleated daughter cells was observed (Figure 2g) that was likely a result of chromosome alignment and segregation errors. Addition of AdΔ19K significantly decreased the frequency of mononucleated cells and consequently increased occurrence of multinucleation in combination-treated cells compared to gemcitabine alone (Figure 2g).

We conclude that following the S-phase arrest, gemcitabine-treated cells went through a prolonged aberrant mitosis and that AdΔ19K infection did not affect the initial drug-induced S-phase arrest nor mitotic duration but promoted mitotic entry, spindle multipolarity, cytokinesis failure and multinucleation. Moreover, AdΔ19K prevented gemcitabine-treated PT45 cells from accumulating in G1 with more cells remaining in the S and G2/M phases, suggesting that combination-treated cells were non-viable and died before entering G1.

### AdΔ19K and DNA-damaging drugs cooperate to increase cellular DNA damage

**Figure 3:**
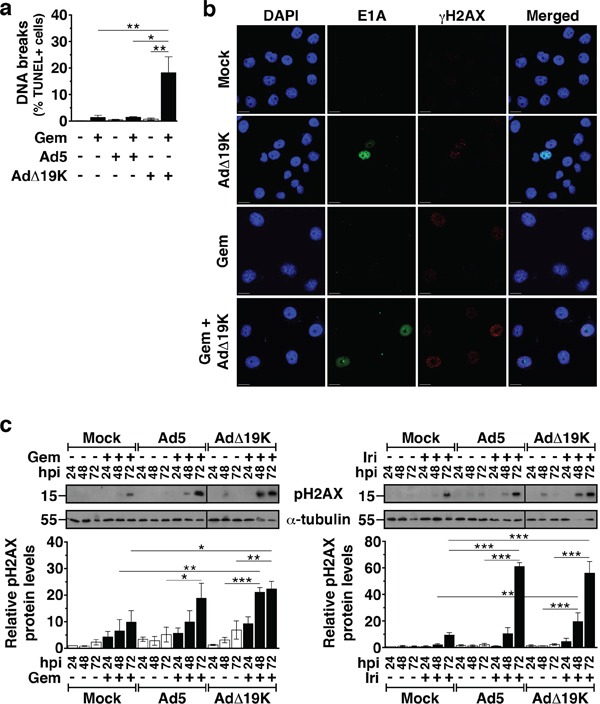
AdΔ19K and DNA-damaging drugs cooperate to induce DNA damage **a.** TUNEL flow-cytometric assay for DNA strand breaks 72hpi in PT45 cells. Error bars represent S.E.M. of 2-4 independent experiments. Only cells with a DNA content of 2-4N were analysed. **b.** Immunofluorescence microscopy analysis of E1A (green) and γH2AX (red) with nuclear DAPI staining (DNA, blue) in PT45 cells fixed at 48hpi using paraformaldehyde. Representative images of 3 independent experiments are shown. Scale bar: 20μm **c.** Immunoblot analysis of phospho-histone H2AX (Ser139) (pH2AX) in PT45 cells, treated with 300ppc viruses ± 5nM gemcitabine (Gem) **(left panel)** and 5μM irinotecan (Iri) **(right panel)**. **Upper panels:** Representative immunoblots of pH2AX (15kDa) with α-Tubulin (55kDa) or Actin (42kDa) as loading controls. Numbers indicate MW size marker (kDa). Vertical lines on the immunoblot indicate points of cropping. **Bottom panels:** pH2AX protein levels were quantified by densitometric analysis, normalised to the loading control and expressed as fold-change relative to mock 24h (=1). Error bars represent S.E.M. of 3 independent experiments. a-c.*.p<0.05 **.p<0.01, ***.p<0.001 (one-way ANOVA with Bonferroni's multiple comparison test).

Despite the accumulation of mitotic aberrations as a consequence of viral infection of gemcitabine-treated cells, the total number of cells in mitosis (<12%) could not account for the significantly increased synergistic cell killing. Both gemcitabine and irinotecan cause DNA damage [31-33] and adenovirus has been reported to induce host cell DNA strand breaks [34, 35]. To investigate whether DNA-damage and repair mechanisms also contributed to the synergistic cell killing, we explored whether AdΔ19K and/or Ad5 enhanced drug-induced DNA-damage. The degree of DNA strand breaks was determined by terminal dUTP nick end labelling (TUNEL) assays and signals were quantified only in cells with a DNA content of 2N-4N, in order to exclude cells with fragmented DNA (apoptotic DNA fragmentation). The frequency of TUNEL-positive cells was significantly elevated in cells treated simultaneously with AdΔ19K and gemcitabine, compared to either agent alone or the combination of Ad5 with gemcitabine (Figure 3a).

To verify the presence of DNA damage, we assessed the expression levels of the DNA damage marker H2AX phosphorylation over time by immunoblotting followed by densitometric quantification. In PT45 cells the combination of AdΔ19K with either gemcitabine or irinotecan caused significantly higher levels of H2AX phosphorylation at 48 and 72h post-treatment, compared to the corresponding single agent treatments (Figure 3c). At 72h, the combination of Ad5 with irinotecan, but not gemcitabine, also induced more phospho-H2AX expression than the single agent treatments (Figure 3c). The increased levels of phospho-H2AX in PT45 cells treated with either gemcitabine or AdΔ19K were observed as distinct foci in cells expressing the viral E1A protein determined by immunofluorescence microscopy (48h; Figure 3b). Similar results were obtained in MIAPaCa-2 cells 48h post-treatment (Figure S3). Therefore, the E1B19K deletion confers earlier and more potent induction of DNA damage in combination with DNA-damaging drugs compared to the intact Ad5 virus.

### AdΔ19K attenuates the DNA damage response induced by gemcitabine and irinotecan

**Figure 4:**
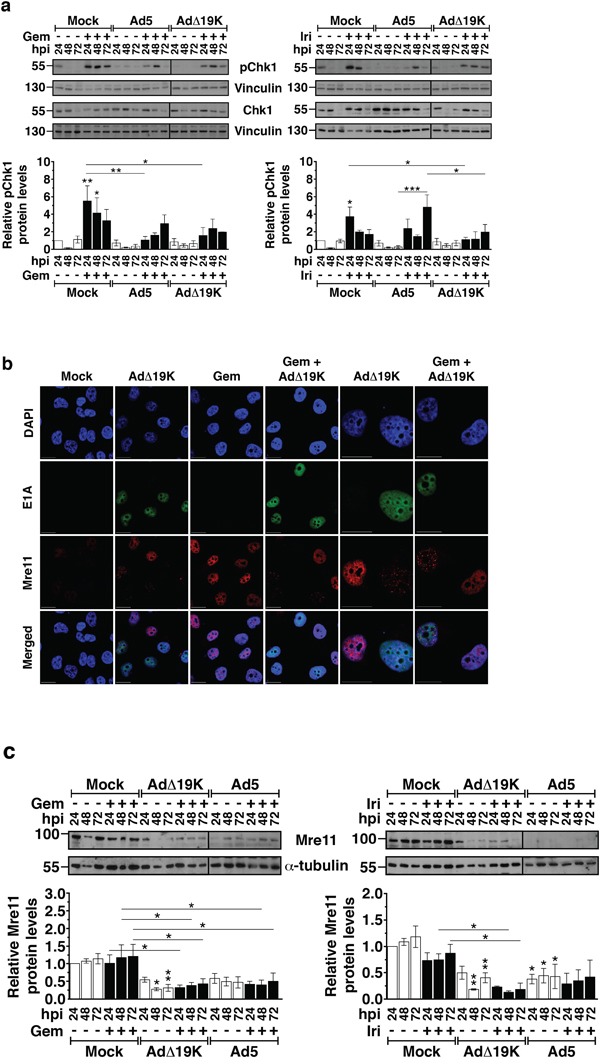
AdΔ19K attenuates the DNA damage response induced by gemcitabine and irinotecan **a.** Immunoblot analysis of phospho-Chk1 (Ser296) (pChk1) and total Chk1 in PT45 cells, treated with 300ppc viruses ± 5nM gemcitabine (Gem) **(left panel)** and 5μM irinotecan (Iri) **(right panel)**. **Upper panels:** Representative immunoblots of phospho- and total Chk1 (56kDa) with Vinculin (130kDa) as loading control. Numbers indicate MW size marker (kDa). Vertical lines on the immunoblot indicate points of cropping. **Bottom panels:** pChk1 protein levels were quantified by densitometric analysis, normalised to total Chk1 and the loading control and expressed as fold-change relative to mock 24h (=1). Error bars represent S.E.M. of 3 independent experiments. **b.** Immunofluorescence microscopy analysis of E1A (green) and Mre11 (red) with nuclear DAPI staining (DNA, blue) in PT45 cells, fixed at 24hpi using paraformaldehyde. Representative images of 2 independent experiments are shown. Scale bar: 20μm. **c.** Immunoblot analysis of Mre11 in PT45 cells, treated with 300ppc viruses ± 5nM gemcitabine (Gem) **(left panel)** and 5μM irinotecan (Iri) **(right panel)**. **Upper panels:** Representative immunoblots of Mre11 (81kDa) with α-Tubulin (55kDa) as loading control. Numbers indicate MW size marker (kDa). Vertical lines on the immunoblot indicate points of cropping. **Bottom panels:** Mre11 protein levels were quantified by densitometric analysis, normalised to the loading control and expressed as fold-change relative to mock 24h (=1). Error bars represent S.E.M. of at least 3 independent experiments. **a-c.** *.p<0.05 **.p<0.01, ***.p<0.001 (one-way ANOVA with Bonferroni's multiple comparison test).

The presence of high levels of DNA damage and the abnormal mitosis led us to investigate drug- and virus-mediated effects on the DNA damage response by determining changes in activation of the checkpoint kinase Chk1. In PT45 cells, gemcitabine and irinotecan induced Chk1 phosphorylation that subsided from 24 to 72h post-treatment (Figure 4a), while in MIAPaCa-2 cells drug-induced Chk1 phosphorylation peaked at 48h (Figure S4a). As expected, no phosphorylation of Chk1 was observed upon infection with either Ad5 or AdΔ19K. Drug-induced Chk1 phosphorylation was overall attenuated in the presence of virus in both cell lines (Figure 4a and S4a). Significantly decreased Chk1 phosphorylation in PT45 cells was observed with AdΔ19K 24h after treatment with either drug, while Ad5 significantly attenuated only gemcitabine-induced Chk1 phosphorylation at 24h (Figure 4a). In MIAPaCa-2 cells, AdΔ19K-mediated attenuation of drug-induced Chk1 phosphorylation was noted 48h after treatment (Figure S4a).

Immediately after adenovirus infection, the DDR is prevented by viral early proteins that act by mislocalising and degrading the MRN complex [23]. To determine whether AdΔ19K also reduced drug-induced Chk1 phosphorylation through the same mechanisms, we examined Mre11 localisation and expression in combination-treated cells. In untreated and gemcitabine-treated cells, diffuse pan-nuclear localisation of Mre11 was observed (Figure 4b). In striking contrast, AdΔ19K-infected cells mislocalised Mre11 in track-like structures and reduced Mre11 expression, regardless of the presence of gemcitabine (right panels, E1A+/Mre11+ cells; Figure 4b). Immunoblotting verified that Mre11 expression was significantly reduced (Figure 4c), consistent with adenovirus-dependent degradation of Mre11. Importantly, the downregulation of Mre11 was still evident in the presence of either gemcitabine or irinotecan (Figure 4c). In PT45 cells infected with AdΔ19K, Mre11 protein levels were significantly lower 24-72h after gemcitabine-treatment and 48-72h after irinotecan-treatment, compared to drug alone (Figure 4c). Significantly lower Mre11 levels were also noted after 48-72h with Ad5 in combination with gemcitabine. In MIAPaCa-2 cells, significant reductions in Mre11 expression compared to drug alone were detected 24-48h post-treatment with AdΔ19K and either gemcitabine or irinotecan, and 24h post-treatment with Ad5 in combination with irinotecan (Figure S4b). To conclude, AdΔ19K mislocalised and degraded Mre11 even in the presence of DNA-damaging drugs, inactivating the MRN complex and consequently attenuating Chk1 activation and the DNA damage and repair signalling.

### AdΔ19K inhibits drug-induced accumulation of Claspin through increased degradation and decreased synthesis

**Figure 5:**
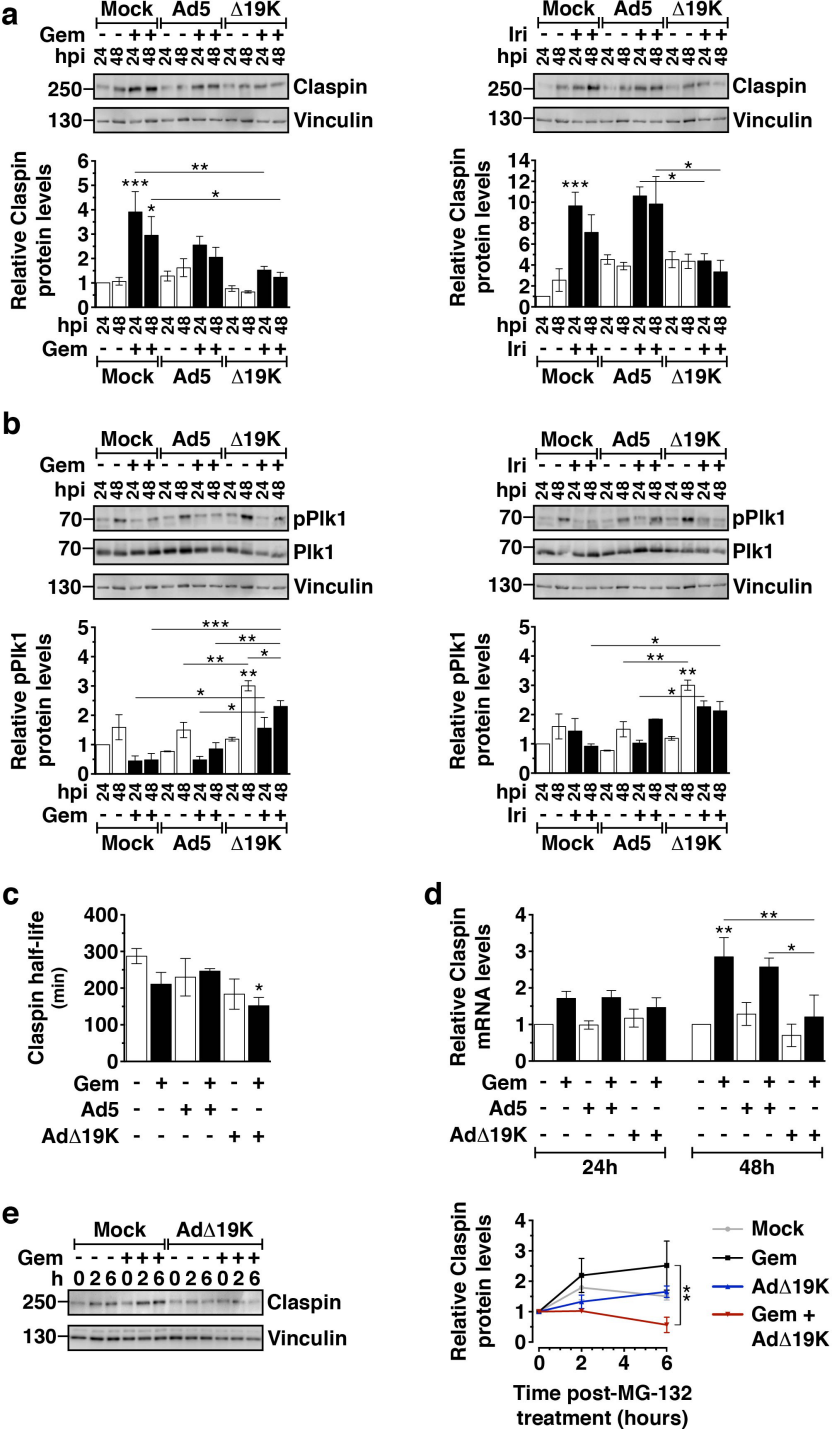
AdΔ19K inhibits drug-induced accumulation of Claspin through increased degradation and decreased synthesis **a.** Immunoblot analysis of Claspin in PT45 cells, treated with 300ppc viruses ± 5nM gemcitabine (Gem) **(left panel)** or 5μM irinotecan (Iri) **(right panel)**. **Upper panels:** Representative immunoblots of Claspin (250kDa) with Vinculin (130kDa) as loading control. Numbers indicate MW size marker (kDa). **Bottom panels:** Claspin protein levels were quantified by densitometric analysis, normalised to the loading control and expressed as fold-change relative to mock 24h (=1). Error bars represent S.E.M. of at least 3 independent experiments. **b.** Immunoblot analysis of phospho-Plk1 (T210) (pPlk1) and total Plk1 in PT45 cells, treated with 300ppc viruses ± 5nM gemcitabine (Gem) **(left panel)** or 5μM irinotecan (Iri) **(right panel)**. **Upper panels:** Representative immunoblots of phospho- and total Plk1 (68kDa) with Vinculin (130kDa) as loading control. Numbers indicate MW size marker (kDa). **Bottom panels:** pPlk1 protein levels were quantified by densitometric analysis, normalised to total Plk1 and the loading control and expressed as fold-change relative to mock 24h (=1). Error bars represent S.E.M. of 2 independent experiments. **c.** PT45 cells were treated with 300ppc of Ad5 or AdΔ19K ± 10nM gemcitabine (Gem). At 48hpi, 3μM of the protein synthesis inhibitor cycloheximide was added to study protein degradation. Cells were harvested at 0, 2, 4 and 6 hours post-cycloheximide treatment and prepared for immunoblot analysis of Claspin expression. Claspin protein levels were quantified by densitometric analysis, normalised to the loading control and expressed relative to the 0h time-point of each treatment (set to 1). The half-life was derived from plotting claspin protein levels against time post-cycloheximide treatment (min) and determining the time at which protein level was at 0.5. Error bars represent S.E.M. of 5 independent experiments. **d.** Claspin mRNA levels measured by qPCR in PT45 cells 24 and 48hpi, normalised to GAPDH internal control and expressed as fold-change relative to mock 24h (=1). Error bars represent S.E.M. of 3 independent experiments. **a-d.** *.p<0.05, **.p<0.01, ***.p<0.001 (one-way ANOVA with Bonferroni's multiple comparison test). **e.** PT45 cells were treated with 300ppc of AdΔ19K ± addition of 10nM gemcitabine (Gem). At 48hpi, 10μM of the proteasome inhibitor MG-132 was added to study protein synthesis. Cells were harvested at 0, 2 and 6 hours post-MG-132 treatment and prepared for immunoblot analysis of Claspin expression. **Left panel:** Representative immunoblot of Claspin (250kDa) with Vinculin (130kDa) as a loading control. Numbers indicate MW size marker (kDa). **Right panel:** Mean values of newly synthesised Claspin protein levels at 0, 2 and 6h post-MG-132 treatment. Claspin protein levels were quantified by densitometric analysis, normalised to the loading control and expressed relative to the 0h time-point of each treatment (set to 1). Error bars represent S.E.M. of 3 independent experiments. **.p<0.01 (two-way ANOVA with Bonferroni's multiple comparison test).

The findings that AdΔ19K promoted drug-induced DNA-damage and attenuated DDR signalling, suggested that the diminished activation of Chk1 played a role in the enhanced cell killing. In response to replication stress or DNA damage, Chk1 is activated by ATR-mediated phosphorylation through recruitment of the adaptor protein Claspin [36, 37]. We found that both gemcitabine and irinotecan induced the accumulation of Claspin at 24-48h post-treatment in PT45 (Figure 5a) and MIAPaCa2 cells (Figure S5a). Interestingly, in PT45 cells both gemcitabine- and irinotecan-induced Claspin accumulation was significantly reduced in the presence of AdΔ19K, but not with Ad5 (Figure 5a). In MIAPaCa-2 cells similar effects were observed in the presence of AdΔ19K (Figure S5a).

For cells to efficiently recover from the ATR/Chk1 checkpoint response and enter mitosis, Claspin needs to be degraded by a mechanism involving the ubiquitin ligase complex β-TrCP-SCF, Aurora-A and Plk-1 [38, 39]. Since we had observed that AdΔ19K-infection of gemcitabine-treated cells decreased pChk1 levels, increased the mitotic index and reduced drug-induced Claspin accumulation, we asked whether virus promoted Claspin degradation. To address this question we examined Plk1 phosphorylation, a major protein responsible for phosphorylating and targeting Claspin for ubiquitination and subsequent proteasomal degradation [39]. In PT45 cells, AdΔ19K, but not Ad5, significantly induced Plk1 phosphorylation 48h post-infection (Figure 5b). No significant changes in pPlk1 were observed in cells treated with gemcitabine alone. In AdΔ19K-infected cells Plk1 phosphorylation was still maintained, albeit to a lesser extent, in the presence of gemcitabine and was significantly higher compared to gemcitabine alone or in combination with Ad5 (Figure 5b). Similar results were obtained with irinotecan (Figure 5b). Analogous to PT45 cells, the presence of AdΔ19K in gemcitabine-treated MIAPaCa-2 cells significantly induced the levels of pPlk1 (Figure S5b). Hence AdΔ19K-induced phosphorylation of Plk1 persisted, albeit to a lesser extent, in the presence of chemotherapeutic drugs. To further investigate whether increased pPlk1 led to Claspin degradation in the presence of AdΔ19K we determined Claspin half-life by cycloheximide-chase assays. The half-life of Claspin was significantly reduced in gemcitabine-treated cells in the presence of AdΔ19K compared to untreated cells (Figure 5c). However, the shorter half-life was unlikely to cause the significant downregulation of Claspin levels observed in combination-treated cells and we explored whether Claspin synthesis was also decreased. Claspin mRNA levels were increased in the presence of gemcitabine (Figure 5d), likely a consequence of drug-induced S-phase arrest. Remarkably, AdΔ19K- but not Ad5-infection of gemcitabine-treated cells significantly decreased Claspin mRNA levels to almost basal after 48h (Figure 5d). In addition, when protein degradation was prevented by the proteasomal inhibitor MG-132 newly-synthesised Claspin accumulated in cells treated with gemcitabine alone but not in cells treated with a combination of gemcitabine and AdΔ19K (Figure 5e). This confirmed that gemcitabine-induced upregulation of Claspin synthesis was inhibited in the presence of AdΔ19K.

### Mre11 and Claspin knockdown enhance cell death and DNA damage induced by AdΔ19K and DNA-damaging drugs

**Figure 6:**
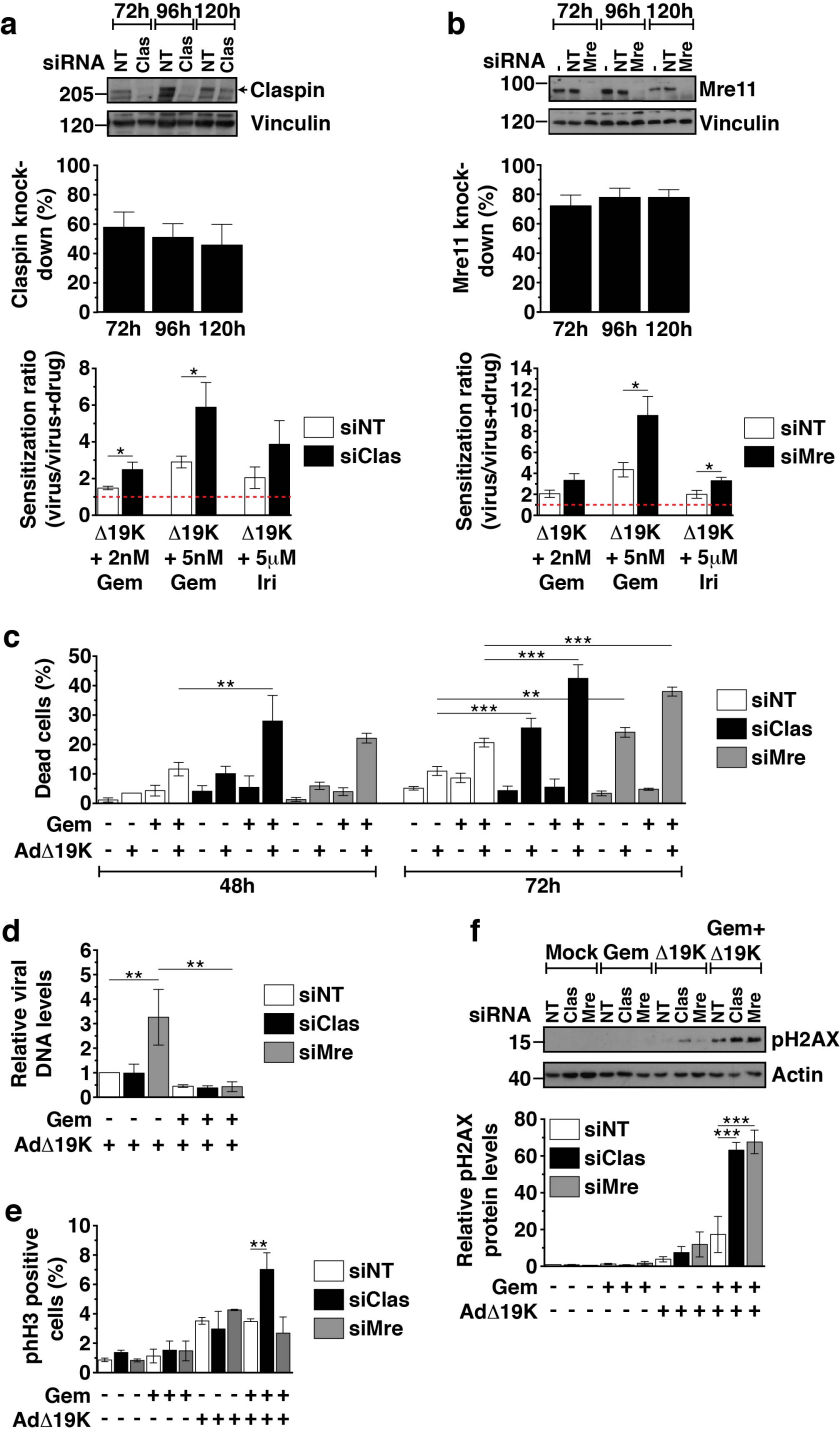
Mre11 and Claspin knockdown enhance cell death and DNA damage in response to AdΔ19K and DNA-damaging drugs PT45 cells were transfected with siRNA against Claspin (siClas), Mre11 (siMre) or non-targeting siRNA (siNT), re-seeded and treated for use in cell viability assays, immunoblotting, Trypan Blue cell death assays, mitotic index analysis and viral genome amplification assays. Untreated cells were harvested at 48, 72, 96 and 120h post-transfection for immunoblot analysis to monitor Claspin and Mre11 knockdown. **a** and **b. Upper panels:** Representative immunoblots of a. Claspin (250kDa) and b. Mre11 (81kDa) with Vinculin (130kDa) as loading control. Numbers indicate MW size marker (kDa). Protein levels were quantified by densitometric analysis, normalised to the loading control and expressed as % protein knockdown relative to siNT. Error bars represent S.E.M. of at least 4 independent experiments. **Bottom panels:** Sensitization ratios (EC_50_ of virus / EC_50_ of virus and drug) derived from cell viability assays 72hpi (corresponding to 120h post-transfection). Error bars represent S.E.M. of at least 4 independent experiments. Drug cytotoxicity (%) ± S.E.M. was: 12.9±4.4% and 27.2±9% with 2nM and 5nM gemcitabine (Gem), respectively, and 18.3±8% with 5μM irinotecan (Iri) in siNT-transfected cells, 8.6±4.5% and 25.2±5.7% with 2nM and 5nM gemcitabine, respectively, and 25±6.5% with 5μM irinotecan in siClas-transfected cells, 29±6.5% and 47.9±1.1% with 2nM and 5nM gemcitabine, respectively, and 42.1±6.1% with 5μM irinotecan in siMre-transfected cells. **c.** Cell death assays using Trypan blue dye incorporation at 48 and 72hpi (corresponding to 96 and 120h post-transfection, respectively). Error bars represent S.E.M. of 3 independent experiments. **d.** Viral genome amplification (Ad-E2A levels) at 48hpi measured by qPCR. Viral DNA was normalized to input DNA (4h) and cellular GAPDH and expressed as fold-change relative to AdΔ19K siNT (=1). Error bars represent S.E.M. of 4 independent experiments. **e.** Mitotic index analysis at 48hpi in unsynchronised PT45 cells stained with propidium iodide, a phospho-histone H3 antibody and an E1A antibody (for identification of infected cells). Dead cells, as identified from their incorporation of FVD, were excluded from the analysis. Error bars represent S.E.M. of 2 independent experiments. **f.** Immunoblot analysis of phospho-histone H2AX (Ser139) (pH2AX) 48hpi in siRNA-transfected PT45 cells treated with 300ppc viruses ± 5nM gemcitabine (Gem). **Upper panel:** Representative immunoblot of pH2AX (15kDa) with Actin (42kDa) as loading control. Numbers indicate MW size marker (kDa). **Bottom panel:** pH2AX protein levels were quantified by densitometric analysis, normalised to the loading control and expressed as fold-change relative to mock 24h (=1). Error bars represent S.E.M. of 3 independent experiments. **a-f.** *.p<0.05, **.p<0.01, ***.p<0.001 (one-way ANOVA with Bonferroni's multiple comparison test).

In order to determine whether the AdΔ19K-dependent downregulation of Claspin and Mre11 in the presence of chemotherapeutic drugs was essential for the enhanced cell killing in response to the combination treatment, we silenced both Claspin and Mre11. Knockdown efficiency in PT45 cells transfected with Claspin or Mre11 siRNA ranged from 46-58% for Claspin and from 72-78% for Mre11, at 72-120h post-transfection (Figure 6a-6b). Silencing of either Claspin or Mre11 increased the sensitization ratios in cells treated with gemcitabine or irinotecan and AdΔ19K, as compared to non-targeting siRNA (Figure 6a-6b; lower panels). We verified that cell death was significantly increased in cells with knockdown of either Claspin or Mre11 in response to the AdΔ19K and gemcitabine combination at 48 and 72h post-treatment, compared to non-targeted knockdown (Figure 6c). In addition, AdΔ19K infection alone but not gemcitabine treatment alone resulted in greater cell death 72h after infection in the knockdown cells (Figure 6c). Interestingly, AdΔ19K DNA replication was increased in cells transfected with Mre11 but not Claspin siRNA (Figure 6d). However, in the presence of gemcitabine, viral replication was greatly attenuated in cells treated with any of the siRNAs (Figure 6d). These data are in agreement with our sensitization studies demonstrating that the increased cell death was not due to enhanced viral replication (Figure 1a-1b, Figure S1a-b).

**Figure 7:**
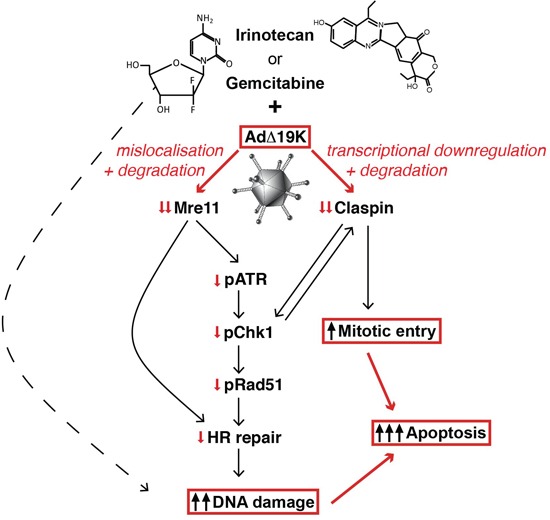
Schematic illustration of cellular mechanisms identified to play major roles in the enhanced cell killing in response to adenovirus and DNA-damaging drugs The cytidine analogue gemcitabine is incorporated into DNA, blocks synthesis and directly causes DNA-strand breaks. Irinotecan, represented by its most potent metabolite SN38, inhibits topoisomerase I preventing completion of DNA-synthesis and causing DNA damage. E1A-expression in AdΔ19K-infected or Ad5-infected cells induces potent expression of E4orf3 and E4orf6 that prevent, together with E1B55K, the cellular DNA-damage response mainly by inactivating Mre11 and rendering the Mre11-Rad50-Nbs1 (MRN) repair complex non-functional (individual viral proteins not shown for simplicity). Degradation and mislocalisation of Mre11 results in direct attenuation of ATR/Chk1 phosphorylation and checkpoint abrogation preventing homologous recombination (HR). In addition, the high expression-levels of E1A/early viral genes in AdΔ19K-infected cells target Claspin for transcriptional downregulation and degradation that prevents full activation of Chk1 and reinforces checkpoint abrogation resulting in significant increases in deregulated entry into mitosis of cells with unrepaired damaged DNA. The end result is high levels of unrepaired DNA damage with synergistic and apoptotic death throughout the cell cycle.

Next we investigated whether Claspin and Mre11 silencing in combination-treated cells was accompanied by increased DNA damage and/or effects on cell-cycle distribution. Cell cycle and mitotic index analysis demonstrated that knocking-down Mre11 had no effect on mitotic index (Figure 6e), while silencing of Claspin significantly increased the mitotic index at 48h, specifically in cells treated with gemcitabine and AdΔ19K (Figure 6e). Importantly, Claspin and Mre11 knockdown resulted in significantly higher phospho-H2AX levels compared to non-targeted knockdown at both 48h and 72h post-treatment with the gemcitabine and AdΔ19K combination (Figure 6f and S6). These results show that downregulation of either Mre11 or Claspin promote both DNA damage and cell death in the presence of concurrent treatment with gemcitabine and AdΔ19K. Taken together, these findings present a mechanism whereby adenovirus cooperates with DNA-damaging drugs to enhance cell killing while still promoting passage through the cell cycle, to complete the viral life cycle despite the high levels of unrepaired cellular DNA damage (Figure 7).

## DISCUSSION

Pancreatic adenocarcinomas are always aggressive with high mortality rates and no effective treatments available for late stage disease. This malignancy has the lowest 5-year survival-rates globally (<5%). To date, no therapy has been reported to significantly prolong survival, including administration of nab-paclitaxel (Abraxane), cisplatin, 5-fluorouracil (5-FU), or gemcitabine [40]. Until now, the cytidine analogue gemcitabine has been the treatment of choice although drug-resistance develops rapidly. Gemcitabine is incorporated into DNA, blocking synthesis and causing DNA-strand breaks [31, 41] and has been evaluated in conjunction with several cytotoxic drugs acting through different mechanisms, including irinotecan that inhibits topoisomerase I [42, 43]. However, only limited improvements in survival, similar to gemcitabine alone, were reported for all combinations and the expected synergistic effects on tumour regression were not observed [40, 42]. Novel therapies with different mechanisms of action to overcome treatment-resistance are therefore urgently needed. Accumulating evidence has shown that oncolytic viruses may fulfil this role by acting on drug-dependent cellular mechanisms to re-sensitize resistant cancers. For example, oncolytic adenoviral mutants enhanced overall responses to gemcitabine in clinical trials for pancreatic cancer [7] and caused regression of pancreatic tumours in xenograft models [10, 44, 45]. We previously reported that our engineered replication-selective mutants, AdΔΔ and AdΔ19K potently and specifically lysed cancer cells with deregulated G1/S cell cycle control and also increased cell killing in combination with DNA-damaging drugs in preclinical models of drug-insensitive pancreatic and prostate cancers [10, 11, 24]. AdΔΔ- or AdΔ19K-infection in conjunction with gemcitabine, irinotecan, cisplatin, or docetaxel resulted in synergistic cell killing in cancer cell lines and prolonged time to tumour progression *in vivo*. The findings supported our hypothesis that deletion of the viral anti-apoptotic gene E1B19K was the cause of the significantly enhanced cell killing, suggesting that AdΔ19K could serve as a tool to identify cellular factors involved in drug-resistance or -sensitization for future therapeutic targeting.

In the current study, we demonstrate that AdΔ19K increases drug-induced DNA-damage and apoptotic death to a greater extent than Ad5, and that virus-mediated checkpoint abrogation plays a key role. We previously demonstrated that suboptimal doses of gemcitabine stimulates viral uptake in pancreatic cancer cell lines [44], which was observed as increased number of E1A-positive cells in the presence of drug in this study. The increased number of virus-infected cells and the early E1A-gene expression in AdΔ19K-infected cells contributes to the enhanced cell killing while viral replication is not required. Increased E1A expression has previously been observed in the absence of the E1B19K gene, resulting in earlier lysis and enhanced viral spread in addition to E1A-induced apoptosis [26, 46-48]. Taken together, our results suggest that E1A also augments drug-induced apoptosis in response to gemcitabine and irinotecan even when the drugs initially attenuate viral replication.

PT45 and MIAPaCa-2 cells have the genetic alterations that characterise pancreatic cancer including activating *KRAS* mutations, *CDKN2A/p16* deletion, and inactivating *TP53* mutations that result in deregulated cell cycle control [49]. Therefore, only limited increases in the S-phase population were noted after virus-infection and no enhancement of drug-induced S-phase arrest was observed, which has been proposed as a potential mechanism of synergy between gemcitabine and oncolytic adenoviruses [50-52]. In contrast, we found that simultaneous infection of gemcitabine-treated cells with either AdΔ19K or Ad5 increased the number of cells in mitosis through G2/M checkpoint abrogation. The combination-treated mitotic cells displayed a high degree of aberrations as a consequence of the extensive unrepaired DNA-damage caused by the drug-induced interruption of DNA synthesis and subsequent strand breaks. Ad5 is a potent inhibitor of the MRN-complex that activates the DNA damage repair response [22, 23, 53, 54]. The inhibition is the result of E1A-induced expression of E4orf3, E4orf6 and E1B55K genes early during infection, targeting Mre11, Nbs1, Rad50 and p53 for sequestration and degradation. In this study, we found that the higher levels of unrepaired DNA damage was caused by adenovirus inactivation of the MRN-mediated repair functions through mislocalization and degradation of Mre11, also in the presence of drugs that induce significant DNA-damage. In agreement with viral hindrance of the Mre11/MRN function, both AdΔ19K- and Ad5-infection decreased the potent activation of pChk1 in drug-treated cells suggesting checkpoint abrogation. Carson et al. demonstrated that mislocalisation of Mre11 by the viral E4orf3 protein was sufficient to prevent ATR signalling, but not concatemirization of viral DNA, which was prevented by E4orf6/E1B55K-mediated targeting of Mre11 for degradation [54]. Furthermore, the E4orf3-dependent mislocalisation of Mre11 reduced ATR/Chk1 signalling in response to the DNA-replication inhibitor hydroxyurea [54]. We conclude that the AdΔ19K-mediated mislocalisation and degradation of Mre11 in the presence of gemcitabine or irinotecan contribute to the attenuation of Chk1 phosphorylation, which subsequently would impair phosphorylation and recruitment of the homologous recombination factor Rad51 to DNA repair foci at stalled replication forks [55] (Figure 7). In addition, Mre11 is also critical for homologous recombination at stalled or collapsed replication forks [56], and its downregulation by AdΔ19K would further attenuate DNA repair resulting in increased accumulation of DNA damage.

Further evidence that the checkpoint was abrogated and cells with significant levels of unrepaired DNA-damage progressed through the cell cycle in combination-treated cells, is provided by our discovery that AdΔ19K prevents drug-induced accumulation of the pChk1/ATR adaptor protein Claspin. AdΔ19K-mediated inhibition of Claspin synthesis and, to a lesser extent, increased degradation, enables checkpoint recovery and mitotic entry even in the presence of high levels of DNA damage. Interestingly, neither Ad5 nor AdΔ19K affected basal Claspin levels while both viruses induced pPlk1. However, only AdΔ19K caused significant inhibition of Claspin expression and increased pPlk1 activation in the presence of gemcitabine or irinotecan. It is possible that the higher levels of early viral genes in AdΔ19K-infected cells result in potent direct E1A- or E1B-binding to transcription-factors that regulate Claspin expression, or that viral E3- or E4-genes interfere with other regulatory elements of Claspin turnover. Both NF-κB and E2F1 were previously reported to regulate Claspin synthesis [57, 58] and interestingly, viral E1A can repress NF-κB-dependent transcription through suppression of IKK activity [59, 60]. We propose that the elevated E1A expression in AdΔ19K-infected cells, followed by increased expression of additional early viral proteins including the E4 products, more potently prevented the accumulation of Claspin and the function of the DNA damage response compared to Ad5. Claspin has previously been reported to be a target of the E7 oncoprotein of human papilloma virus (HPV)-16 that increased the proteasomal degradation by deregulating components of the Aurora-A/Plk1/SCF^β-TrCP^ degradation machinery, thereby attenuating DNA damage responses and promoting mitotic entry [61]. Also, hepatitis B virus (HBV) X protein was shown to mediate Plk1 activation, inducing Claspin degradation and attenuating both DNA repair and the checkpoint responses, thereby resulting in cell cycle progression and eventual death [62]. However, to our knowledge, adenovirus-mediated inhibition of Claspin activity had not been previously reported. Our findings reveal a potential novel mechanism whereby adenovirus destabilises Claspin, relaxes S-G2/M checkpoint activation, forces progression through the cell cycle in the presence of DNA damage and ultimately augments cell killing. It will be of great interest to determine whether adenovirus-mediated destabilisation of Claspin recruits similar mechanisms to those reported for HPV or HBV. Another possibility is that adenovirus-mediated disruption of PP2A phosphatase activity by the viral E4orf4 protein [63, 64] could stabilize pPlk1 thereby inducing Claspin degradation, as PP2A was shown to de-phosphorylate Plk1 in response to DNA damage [65].

Importantly, knockdown of Claspin or Mre11 enhanced the cell death in combination-treated cells, strongly supporting our evidence for AdΔ19K-mediated downregulation of Claspin and Mre11 as major causes for the enhanced cell death (Figure 7). Mre11 knockdown potentiated DNA damage in combination-treated cells but had no effect on cell cycle distribution or viral DNA replication. We conclude that downregulation of Mre11 contributes to the observed apoptotic cell death that occurred throughout the cell cycle. Knockdown of Claspin also increased DNA damage in combination-treated cells, with no effects on viral DNA replication, however the number of cells entering mitosis increased. These results suggest that the enhanced cell death observed when Claspin is downregulated might be a result of both increased DNA damage and mitotic entry in combination-treated cells, with subsequent mitotic aberrations.

In conclusion, our data strongly point toward a role for the potent earlier E1A expression in the absence of the E1B19K-gene, in promoting the expression of the viral E1B and E4 genes that attenuate the DNA damage response, as essential for the synergistic cell killing. Although less pronounced, mitotic aberrations and DNA damage were also observed with Ad5 infection. However, AdΔ19K greatly attenuated drug-induced Claspin expression, which was not significantly reduced in the presence of Ad5. We suggest that both aberrant mitosis and enhanced apoptotic death throughout the cell cycle due to high levels of DNA damage is required for the synergistic cell killing. Our findings have revealed novel cellular targets that are deregulated by adenovirus to subvert the cellular defence against both viruses and other cytotoxic agents. We propose that exploiting these factors in combination with DNA-damaging drugs, improved anti-cancer therapeutics could be developed resulting in greatly enhanced tumour cell killing. Oncolytic adenoviruses could be designed to synergise with DNA-damaging drugs by incorporating the E1B19K-deletion in combination with inhibitors or si/shRNA that target pChk1/Claspin and Mre11/Rad51.

## MATERIALS AND METHODS

### Cell lines and culture conditions

The human pancreatic adenocarcinoma cell lines PT45 (Prof H. Kalthoff, Kiel, Germany) and MIAPaCa-2 (ATCC, VA) are derived from primary PDAC tumours. The cell lines were STR-profiled (LGC Standards, UK and Cancer Research UK) and verified to be identical to the profiles reported by the suppliers and to the original vial. Cells were grown at 37°C and 5% CO_2_ in Dulbecco modified Eagle's medium (DMEM) supplemented with 10% Fetal Bovine Serum (FBS) and 1% penicillin and streptomycin (Penicillin 10000 units/ml, Streptomycin 10mg/ml; P/S) (all purchased from Sigma-Aldrich, MO). DMEM contained 4.5g/L glucose, L-glutamine, sodium pyruvate and sodium bicarbonate. In all experiments cells were seeded in 10% FBS/1% P/S DMEM.

### Viruses and infections

Both wild-type virus Ad5 and the mutant AdΔ19K were generated from the species C wild-type adenovirus type 5 plasmid pTG3602 (a gift from Dr Majid Mehtali, Transgéne, Strasbourg, France), produced, purified and characterised as previously described [11, 24]. AdΔ19K is deleted in the anti-apoptotic E1B19K gene (AdΔ19K). The viral particle (vp) to infectious units (plaque-forming units; pfu) was 28 and 12 vp/pfu for Ad5 and AdΔ19K, respectively. All infections were performed in serum-free DMEM −/+ the indicated doses of viruses and 2h later the medium was replaced with 10% FBS/1% P/S DMEM −/+ the indicated dose of drug(s).

### Trypan blue inclusion cell death assay

PT45 and MIAPaCa-2 cells were infected with 300ppc of Ad5 or AdΔ19K −/+ addition of 5nM (PT45) or 20nM (MIAPaCa-2) gemcitabine (Gemzar; Eli Lilly, Indianapolis, IN) or 5μM irinotecan (Campto, Hospira UK Limited, Leamington Spa, UK). At the indicated times cells were trypsinised and cell suspension was mixed with 0.4% Trypan Blue dye (Bio-Rad Laboratories, Inc, CA) at 1:1 ratio and 10μl in duplicates were loaded onto a dual-chambered counting slide (Bio-Rad). Cell count and viability were assessed using a TC20™ automated cell counter (Bio-Rad). Percentage cell viability was recorded and used to calculate cell death.

### Cell viability assays

Cell viability assays were performed as described previously [11]. Cells were infected with Ad5 or AdΔ19K −/+ addition of gemcitabine or irinotecan in 2% FBS/1% P/S DMEM. Drug concentration was fixed at doses previously determined to kill 20-40% of cells. Cells were assayed 72h later using the 3-(4,5-dimethylthiazol-2-yl)-5-(3-carboxymethoxyphenyl)-2-(4-sulfophenyl)-2H-tetrazolium assay (Promega, Southampton, UK) to quantify live cells as an indirect measurement of cell death. Dose–response curves were generated to determine the concentration of each agent killing 50% of cells (EC_50_) using untreated cells or cells treated with one agent only as controls. Each data point was generated from triplicate samples and experiments repeated at least three times.

### Cleaved caspase-3 apoptotic assay

PT45 cells were infected with 300ppc of Ad5tg or AdΔ19K −/+ addition of 5nM gemcitabine. Where indicated, 25μM of the pan-caspase inhibitor Calbiochem^®^ Z-VAD(OMe)-FMK (5mM in dimethyl sulfoxide (DMSO); Millipore, MA) was added simultaneously with gemcitabine or staurosporine. Mock-infection and 16h treatment at 1μM with the apoptosis-inducing agent staurosporine (1mM in DMSO; Sigma-Aldrich), were used as negative and positive controls, respectively. At 72hpi cells were harvested, fixed and stained using the FITC Active Caspase-3 Apoptosis Kit (BD Biosciences Pharmingen, CA) according to the manufacturer's guidelines. Additionally, cell pellets were re-suspended in 250μl of PBS containing 50μg/ml of propidium iodide (PI) (1mg/ml in water; Thermo Fisher Scientific, CA) and 100μg/ml of ribonuclease A (RNAse A) (33mg/ml in Tris-HCl/glycerol; Sigma-Aldrich) and incubated for 30min at 22°C. Flow-cytometric data acquisition was performed using BD CellQuest™ software operated on a BD FACSCalibur instrument (both from Becton Dickinson, NJ). PI and FITC signals were detected in the FL-3 and FL-1 channel, respectively, of the 488nm argon laser. Dot plots of Side Scatter (SSC-H) vs Forward Scatter (FSC-H) were used to exclude debris, followed by doublet exclusion using the area and width of the FL2 channel. Acquisition stopped when 20000 events were acquired in the doublet-exclusion gate. Cell-cycle specific apoptosis was measured by plotting the FL1-H channel, where FITC was detected, against the FL3-H channel, where PI was detected, and gates for apoptotic cells with DNA content of 2N (G1-phase), 2-4N (S-phase), 4N (G2/M-phase) and >4N (polyploid), where applied. Post-acquisition data analysis was performed using the FlowJo v7.6.5 software (Tree star, Inc, OR).

### Cell synchronisation

PT45 cells were treated with 2.5mM thymidine (100mM in water; Alfa Aesar, MA) for synchronisation in early S-phase. 24h after treatment, cells were released from the thymidine block by washing twice with PBS. Cells were immediately infected with 300ppc of viruses −/+ 5nM gemcitabine.

### Cell cycle and mitotic index analysis

PT45 cells were infected with 300ppc of viruses −/+ 5nM gemcitabine. At the indicated times post-infection, supernatant and cells were harvested, washed with PBS and incubated with 250μl of the fixable viability dye (FVD) eFluor^®^ 506 (eBioscience, CA) diluted 1:1000 in PBS. Following a 30min incubation at 4°C, cells were washed in PBS and fixed with cold 70% ethanol (30min, 4°C). All centrifugations henceforth were performed at 2000rpm for 3min. Cells were washed with 1ml of 1% FBS/PBS and permeabilised using cold 0.25% Triton X-100 diluted in 1% FBS/PBS. Following a 10min incubation at 4°C, cells were centrifuged and incubated in 100μl of rabbit polyclonal anti-phospho-histone H3 (S10) (Abcam, UK) and mouse monoclonal anti-E1A antibodies diluted in 1% FBS/PBS (30min, 22°C). Following two washes in 2ml of 1% FBS/PBS, cells were incubated in 100μl of goat anti-rabbit Alexa Fluor^®^ 488 and anti-mouse Alexa Fluor^®^ 647 IgG (H+L) antibodies (Life Technologies, Thermo Fischer Scientific, UK) diluted in 1% FBS/PBS (30min, 22°C). Cells were then washed with 3ml of 1% FBS/PBS and incubated in 200μl of PI (50μg/ml)/RNAse A (100μg/ml) solution (30min, 22°C). Cells were immediately acquired using the BD FACSDiva™ software operated on the BD LSRFortessa™ cell analyzer (both from Becton Dickinson). PI and Alexa Fluor^®^ 488 signals were detected in the B695/40 and B5300/30 filters, respectively, of the 488nm blue laser. FVD eFluor^®^ 506 and Alexa Fluor^®^ 647 signals were detected in the V525/50 filter of the 405nm violet laser and R670/14 filter of the 640nm red laser, respectively. Cell debris and doublets were excluded, followed by gating of live cells and acquisition of 20000 events. Data were analyzed using the FlowJo v7.6.5 software.

For cell cycle analysis with PI only, PT45 and MIAPaCa-2 cells were infected, treated and harvested as specified and fixed with cold 70% ethanol (30min, 4°C). Cells were pelleted, washed with 1ml of PBS and incubated in 300μl of PI (50μg/ml)/RNAse A (100μg/ml) solution (30min, 22°C). Immediately after staining, cells were analysed using a BD FACSCalibur instrument. Cell debris and doublets were excluded and 20000 events were acquired. PI fluorescence was detected using the FL3 channel of the 488nm argon laser. Data were analyzed using the FlowJo v7.6.5 software.

### Immunofluorescence microscopy analysis

PT45 cells were seeded on coverslips (Menzel-Gläser, Germany), treated as specified and at the indicated times post-infection processed as follows:

### Methanol fixation method

Cells were fixed in 100% ice-cold methanol (20min, 4°C), followed by 20min blocking in 5% FBS/PBS and incubation (overnight, 4°C) in mouse monoclonal anti-Aurora-A (IAK-1) (BD Biosciences) and rabbit polyclonal anti-α-Tubulin (Abcam) antibodies diluted in 5% FBS/PBS. Cells were washed three times in PBS and incubated (1h, 22°C) in goat anti-mouse AlexaFluor^®^ 488 and anti-rabbit AlexaFluor^®^ 594 IgG (H+L) antibodies (both from Life Technologies) diluted in 5% FBS/PBS.

### Paraformaldehyde fixation method

Cells were fixed (10min, 22°C) in 4% paraformaldehyde/PBS (made from 16%(w/v) formaldehyde; TAAB, UK), followed by permeabilization (10min, 22°C) in Triton buffer (0.5% Triton X-100, 20mM Hepes KOH pH7.9, 50mM NaCl, 3mM MgCl2, 300mM Sucrose). Cells were blocked in 0.05% Tween-20/3% BSA/PBS (15min, 22°C) and incubated (overnight, 4°C) in primary antibodies diluted in blocking buffer. Antibodies used: mouse monoclonal anti-Ad-DBP (37.3) (a gift from K. Benihoud, Institut Gustave Roussy, Villejuif, France), rabbit polyclonal anti-phospho-histoneH2A.X (Ser139) (Cell Signalling Technology, MA), mouse monoclonal anti-E1A and/or rabbit polyclonal anti-Mre11 (Genetex Inc., CA). Coverslips were washed twice in blocking buffer and incubated (1h, 22°C) in secondary antibodies (goat anti-mouse or anti-rabbit AlexaFluor^®^ 594 and/or goat anti-mouse AlexaFluor^®^ 488) diluted in blocking buffer.

### Mounting and analysis

Coverslips were washed in PBS and distilled water and were allowed to dry (30min, 22°C) before mounting on slides (ESCO Optics, NJ) using the ProLong Gold antifade reagent with DAPI (Life Technologies, Thermo Fisher Scientific). Slides were analysed using a Zeiss Axioplan epifluorescent microscope. Images were acquired using the confocal laser scanning microscope Zeiss LSM510 META.

### PT45 histone H2B-mCherry stable cell line generation and time-lapse microscopy

PT45 cells were transfected with 5μg of histoneH2B-mCherry construct (a gift from Dr Spiros Linardopoulos, The Institute of Cancer Research, London, UK) using 16μl Lipofectamine™ 2000 (Life Technologies Thermo Fisher Scientific), according to the manufacturer's instructions. 24h later cells were harvested, washed in PBS and fluorescently sorted using the BD FACSAria™ cell sorter (Becton Dickinson) based on mCherry expression (detected in the YG610/20-A filter of the yellow-green 561nm laser). The PT45 histoneH2B-mCherry cells were fluorescently sorted several times until more than 90% of cells expressed mCherry. For time-lapse microscopy PT45 histoneH2B-mCherry cells were synchronised and infected as detailed above. 2hpi the medium was replaced with 10% FBS/Leibovitz's L15 medium (Life Technologies) −/+ 5nM gemcitabine. 24hpi cells were subjected to a 72h time-lapse imaging by phase-contrast and fluorescence microscopy using a Zeiss Axiovert 200M fluorescence microscope. Images from 3 different fields per condition were acquired every 15min from 24 to 96hpi. Data were analysed using AxioVision Rel. 4.9.1 (Carl Zeiss, Germany) and NIH ImageJ software.

### TUNEL assay

PT45 cells were infected with 300ppc of viruses −/+ 10nM gemcitabine. 72hpi cells were harvested, fixed and stained using the APO-BrdU kit (BD Biosciences Pharmingen) according to the manufacturer's guidelines. Flow-cytometric data acquisition was performed using BD CellQuest™ software operated on a BD FACSCalibur instrument (both from Becton Dickinson). FITC fluorescence was detected in the FL-1 of the 488nm argon laser. Cell debris and doublets were excluded and 20000 events were acquired. Post-acquisition data analysis was performed using the FlowJo v7.6.5 software.

### Immunoblotting

PT45 and MIAPaCa-2 cells were infected and treated as specified, and harvested at the indicated times. Cells were lysed in RIPA buffer (50mM Tris pH 8.0, 150mM NaCl, 1% Triton X-100, 0.5% sodium deoxycholate, 0.1% SDS) supplemented with protease inhibitor cocktail and PhosSTOP phosphatase inhibitor (Roche Diagnostics, Switzerland). Cell lysates were incubated on ice (15min), centrifuged (15min, 16100xg, 4°C) and quantitated for protein using the Bio-Rad Protein assay (Bio-Rad) according to the manufacturer's guidelines. Cell lysates were mixed with 2X sample Laemmli buffer (0.125M Tris-HCl pH6.8, 20% glycerol, 4% SDS, 0.01% bromophenol blue and freshly-added 10% β-mercaptoethanol) and incubated for 5min at 95°C. Equal amounts (typically 30-60μg) of protein were resolved by SDS Polyacrylamide Gel Electrophoresis (SDS-PAGE) using the Mini-PROTEAN^®^ Tetra cell system (Bio-Rad). Proteins were then transferred to nitrocellulose membranes (Hybond-ECL, GE Healthcare, UK) using the Trans-Blot^®^ SD semi-dry electrophoretic transfer cell (Bio-Rad) or the Mini Trans-Blot^®^ electrophoretic transfer cell system (Bio-Rad). Membranes were incubated (overnight, 4°C) in the following primary antibodies: rabbit polyclonal anti-phospho-histone H2A.X Ser139, rabbit polyclonal anti-α-Tubulin, goat polyclonal anti-Actin (C-11) (SantaCruz Biotechnology, Inc; TX), rabbit polyclonal anti-phospho-Chk1 Ser296 (133D3) (Cell Signalling Technology), rabbit polyclonal anti-Chk1 (Cell Signalling Technology), mouse monoclonal anti-Vinculin (SPM227) (Abcam), rabbit polyclonal anti-Mre11, rabbit polyclonal anti-Claspin (Cell Signalling Technology), rabbit polyclonal anti-phospho-Plk1 Thr210 (Enzo Life Sciences, UK), mouse monoclonal anti-Plk1 (Abcam) and mouse monoclonal anti-β-Tubulin (SAP.4G5) (Abcam). Membranes were washed in 0.1% Tween-20/TBS, and incubated (1h at 22°C or 2h at 4°C) in polyclonal anti-goat, mouse or rabbit immunoglobulins/HRP (Dako). Immunodetection was performed using enhanced chemiluminescence substrate ECL and ECL-Plus (PerkinElmer). Protein bands were visualised on X-ray films (FujiFilm) or using the G:Box iChemi-XT imaging system (Syngene, UK) and quantified by densitometric analysis using the NIH ImageJ software.

### mRNA analysis by reverse transcriptase qPCR

PT45 cells were infected with 300ppc of viruses, treated with 5nM gemcitabine and harvested (excluding supernatant) at the indicated times. RNA was extracted using the RNeasy Mini Kit (Qiagen, Netherlands) according to the manufacturer's instructions and purified from contaminating DNA using the DNA-free™ kit (Ambion^®^, Life Technologies, CA) according to the manufacturer's instructions. 1μg of RNA was reversed transcribed in a 50μl reaction containing 1x TaqMan^®^ Reverse Transcription Buffer, 5.5mM MgCl_2_, 500μM deoxyNTPs mixture, 2.5μM random hexamers, 0.4units/μl RNase inhibitor and 1.25units/μl MultiScribe^®^ Reverse Transcriptase (all from Applied Biosystems, Thermo Fisher Scientific). Reverse transcription was performed using a DNA Engine Dyad^®^ Peltier thermal cycler (MJ Research) by incubating at 25°C for 10min, followed by 30min incubation at 48°C and a 5min incubation at 95°C. 20ng of complementary DNA (cDNA) were used in qPCR analysis of viral E1A and Penton genes and the cellular Claspin with GAPDH as internal control. qPCR using the standard curve method (Applied Biosystems 7500 Instrument) was performed with SYBR^®^ Green PCR master mix (Applied Biosystems) and 200nM of the following primers: E1A 5′-TGCCAAACCTTGTACCGGA-3′ (forward) and 5′-CGTCGTCACTGGGTGGAAA-3′ (reverse), Penton 5′-GATCGGAAAACCTCTCGAGAAA-3′ (forward) and 5′-CGTAGGAGGGAGGAGGACCTT-3′ (reverse), Claspin 5′-ACAGTGATTCCGAAACAGA-3′ (forward) and 5′-TGCTCCTCGGCACTGTCATA-3′ (reverse). Melting (dissociation) curves for each primer set were generated for primer quality control.

### siRNA transfections

PT45 cells were left untransfected or transfected with 25nM of siGENOME non-targeting (NT) siRNA #1 control (D-001210-01-05), siGENOME SMARTpool Claspin (CLSPN) siRNA or siGENOME SMARTpool MRE11A siRNA, using DharmaFECT1 transfection reagent (all purchased from Dharmacon, GE Healthcare) according to the manufacturer's instructions. siRNA sequences: siGENOME SMARTpool CLSPN (D-005288-01/02/03/04) ‘5-GGAAAUACCUGGAGGAUGA-3’, ‘5-GCAGAUGGGUUCUUAAAUG-3’, ‘5-GGACG UAAUUGAUGAAGUA-3’, ‘5-GAAUUUAUAUGCUG GGAAA-3’ and siGENOME SMARTpool MRE11A (D-009271-01/02/03/04) ‘5-GAUGAGAACUC UUGGUUUA-3’, ‘5-GAAAGGCUCUAUCGAAUGU-3’, ‘5-GCUAAUGACUCUGAUGAUA-3’, ‘5-GAGUAUA GAUUUAGCAGAA-3’. After 6h medium was replaced with 10% FBS/1% P/S DMEM. 24-32h later non-transfected, siNT-, siCLSPN- and siMRE11-transfected cells were harvested, counted and re-seeded in 96-well plates for use in cell viability assays or 6-well plates for use in immunoblotting, trypan blue cell death assays, mitotic index analysis and/or viral genome amplification assays.

### Viral genome amplification

PT45 cells were infected with 300ppc of viruses, treated with 5nM gemcitabine and harvested (excluding supernatant) at 4, 24, 36, 48 and 72hpi. Cell suspension was pelleted, snap-frozen and stored at −80°C. DNA was extracted using the QIAamp DNA Blood Mini Kit, according to the manufacturer's instructions (Qiagen) and used for quantitative PCR (qPCR) analysis as previously described [10].

### Quantification of E1A protein expression by flow cytometry

PT45 and MIAPaCa-2 cells were infected with 300ppc (PT45) or 100ppc (MIAPaCa-2) of viruses −/+ 5nM (PT45) or 10nM (MIAPaCa-2) gemcitabine. At the indicated times cells were harvested (excluding supernatant) and 0.5-1×10^6^ cells/ml were resuspended in ice-cold 3% BSA/1% sodium azide/PBS, pelleted and fixed in 0.5ml 100% methanol (10min, −20°C). Cells were washed twice in 1% BSA/PBS and permeabilised in 0.5ml of 0.5% Triton-X100/PBS (15min, 22°C), followed by wash in 0.1% Triton/PBS and incubation with mouse monoclonal anti-E1A (M58) antibody (Labvision, Thermo Fisher Scientific) diluted in 3% BSA/PBS (30min, 22°C). Cells were washed once with PBS and incubated with anti-mouse monoclonal FITC-conjugated antibody (Dako, Denmark) diluted in 3% BSA/PBS (30min, 22°C). Cells were washed twice in PBS, resuspended in 3% BSA/1% sodium azide/PBS and analysed by flow cytometry using a BD FACSCalibur instrument. Cell debris was excluded and 20000 events were acquired. FITC was detected using the FL1 channel of the 488nm argon laser. Data were analyzed using the FlowJo v7.6.5 software.

## SUPPLEMENTARY FIGURES AND TABLES


