# Supplementary material for: The E1B19K-deleted oncolytic adenovirus mutant AdΔ19K sensitizes pancreatic cancer cells to drug-induced DNA-damage by down-regulating Claspin and Mre11
Source: Oncotarget. 2016 Feb 10;7(13):15703–24. doi: 10.18632/oncotarget.7310 (PMC4941271; doi:10.18632/oncotarget.7310)
Supplement: Supplementary file 1 [file oncotarget-07-15703-s001.pdf]

## SUPPLEMENTARY FIGURES AND TABLES

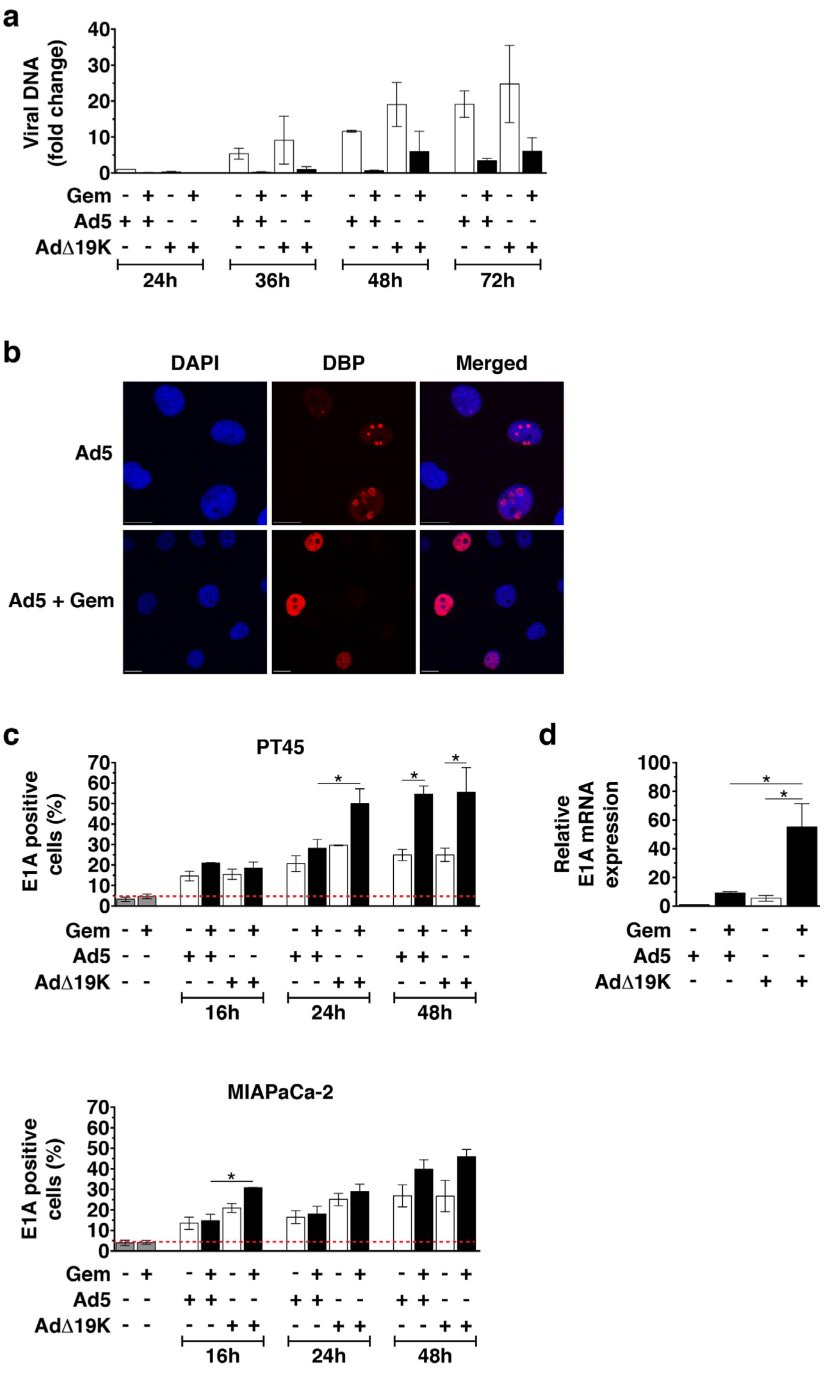


**Supplementary Figure S1: The enhanced cell killing is not due to drug-mediated increases in viral replication. a.** Viral genome amplification (Ad-E2A levels) in PT45 cells determined by qPCR. Viral DNA was normalized to input DNA (4h) and cellular GAPDH and expressed as fold-change relative to Ad5 24h (=1). Error bars represent S.E.M. of 2 independent experiments. **b.** Immunofluorescence microscopy analysis of the adenovirus DNA binding protein (DBP, red) with nuclear DAPI staining (DNA, blue) in Ad5-infected PT45 cells (100ppc) ± 10nM gemcitabine (Gem), fixed at 24hpi using parafolmaldehyde. Representative images of 2 independent experiments are shown. Scale bar: 20μm. **c.** Adenovirus E1A protein expression measured by flow cytometry in PT45 **(top panel)** and MIAPaCa-2 **(bottom panel)** cells. Red dotted lines indicate baseline (background) fluorescence. Error bars represent S.E.M. of 3-4 independent experiments. **d.** Adenovirus E1A mRNA levels measured by qPCR in PT45 cells 24hpi and expressed as fold-change relative to Ad5 (=1). E1A mRNA levels were normalised to adenovirus penton and GAPDH internal control to determine E1A expression as a function of viral late gene expression (indicative of replication). Error bars represent S.E.M. of 3 independent experiments. **a-d.** *.$p<0.05$ (one-way ANOVA with Bonferroni's multiple comparison test).

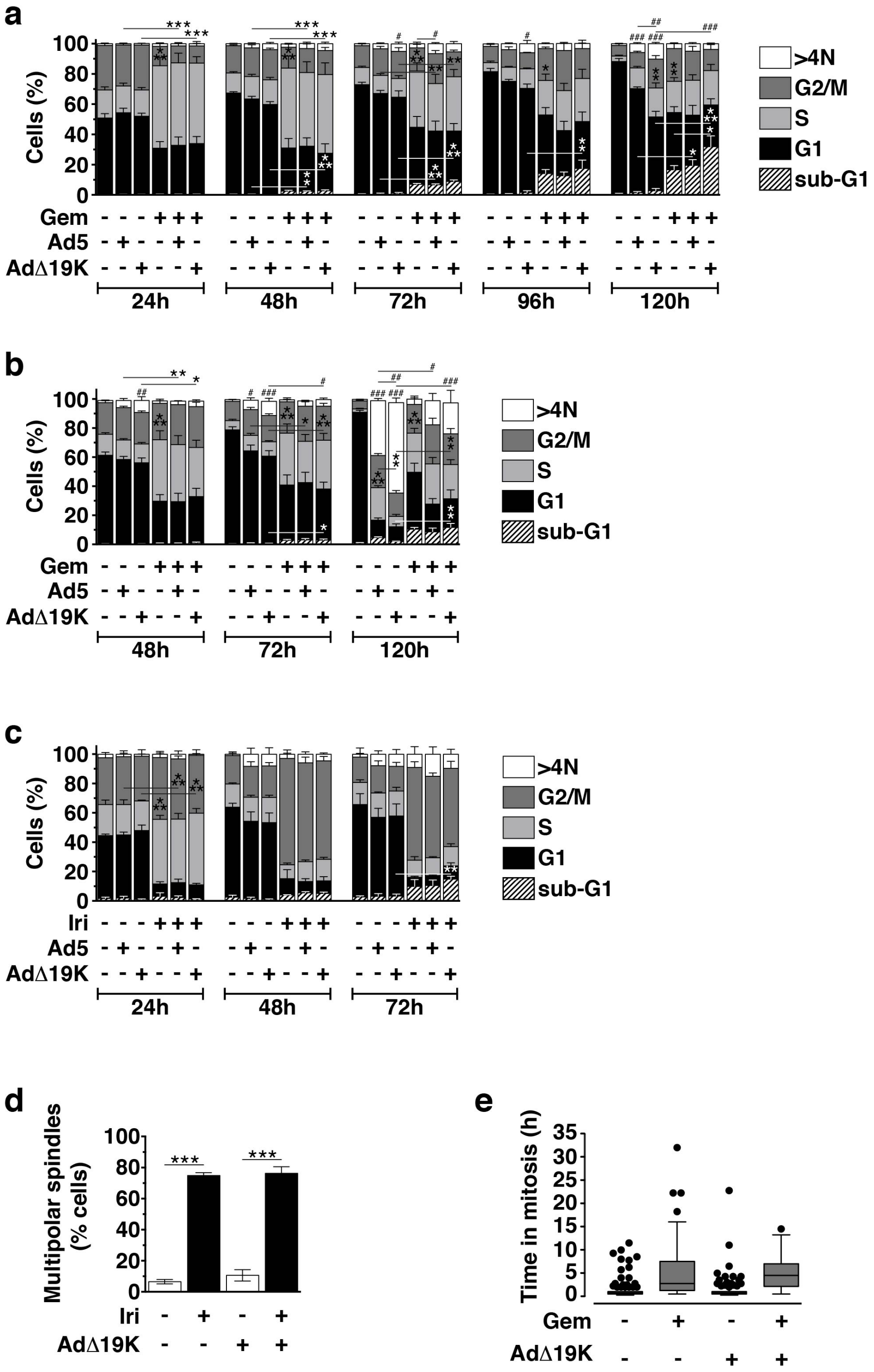


**Supplementary Figure S2: Cell cycle analysis in unsynchronised cells. a-c.** Cell cycle analysis in unsynchronised PT45 (A and C) and MIAPaCa-2 (B) cells, treated with 100ppc (PT45) or 300ppc (MIAPaCa-2) of viruses ± 10nM gemcitabine (Gem) or 12.5µM irinotecan (Iri), fixed at the indicated times post-infection and stained with PI. White asterisks, black asterisks and hashtags represent statistical significance in subG1, S-phase and >4N cell population, respectively. Error bars represent S.E.M. of at least 3 independent experiments. **d.** Quantification of spindle multipolarity in PT45 cells infected with 300ppc of AdΔ19K ± 5µM irinotecan (Iri), fixed at 48hpi and subjected to immunofluorescence microscopy analysis of Aurora-A and α-Tubulin with nuclear DAPI staining. At least 100 mitotic cells were counted/condition/study. Error bars represent S.E.M of 3 independent experiments. **e.** Time-lapse microscopy 24-96hpi in PT45 cells stably expressing histone H2B-mCherry. Tukey's box-and-whisker plots (with whiskers spanning 1.5 x IQR and outliers shown) showing the duration of mitosis, defined from the time of nuclear envelope breakdown until the time of sister chromatid separation. Mitotic cells from each independent experiment were pooled together. At least 100 mitotic cells were analysed. **a-d.** *.p<0.05, **.p<0.01, ***. p<0.001 (one-way ANOVA with Bonferroni's multiple comparison test).

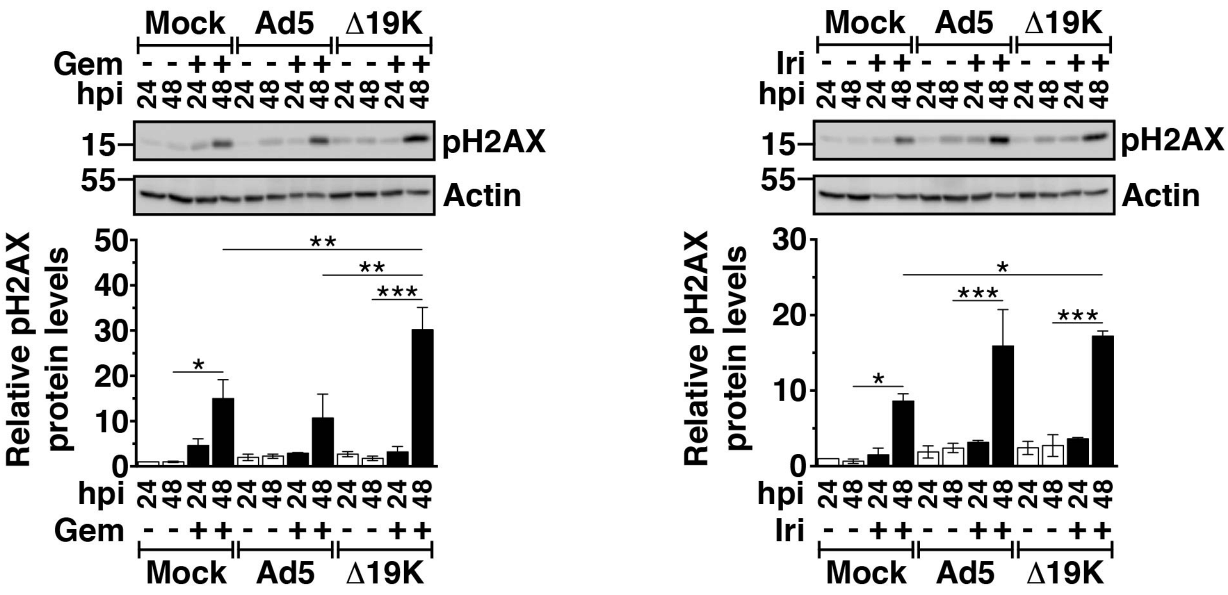


**Supplementary Figure S3: AdΔ19K and DNA-damaging drugs cooperate to induce DNA damage in MIAPaCa-2 cells.** Immunoblot analysis of phospho-histone H2AX (Ser139) (pH2AX) in MIAPaCa-2 cells treated with 300ppc viruses ± 20nM gemcitabine (Gem) **(left panel)** and 15µM irinotecan (Iri) **(right panel)**. **Upper panels:** Representative immunoblots of pH2AX (15kDa) with Actin (42kDa) as loading control. Numbers indicate MW size marker (kDa). **Bottom panels:** pH2AX protein levels were quantified by densitometric analysis, normalised to the loading control and expressed as fold-change relative to mock 24h (=1). Error bars represent S.E.M. of 2 independent experiments. *.p<0.05 **.p<0.01, ***.p<0.001 (one-way ANOVA with Bonferroni's multiple comparison test).

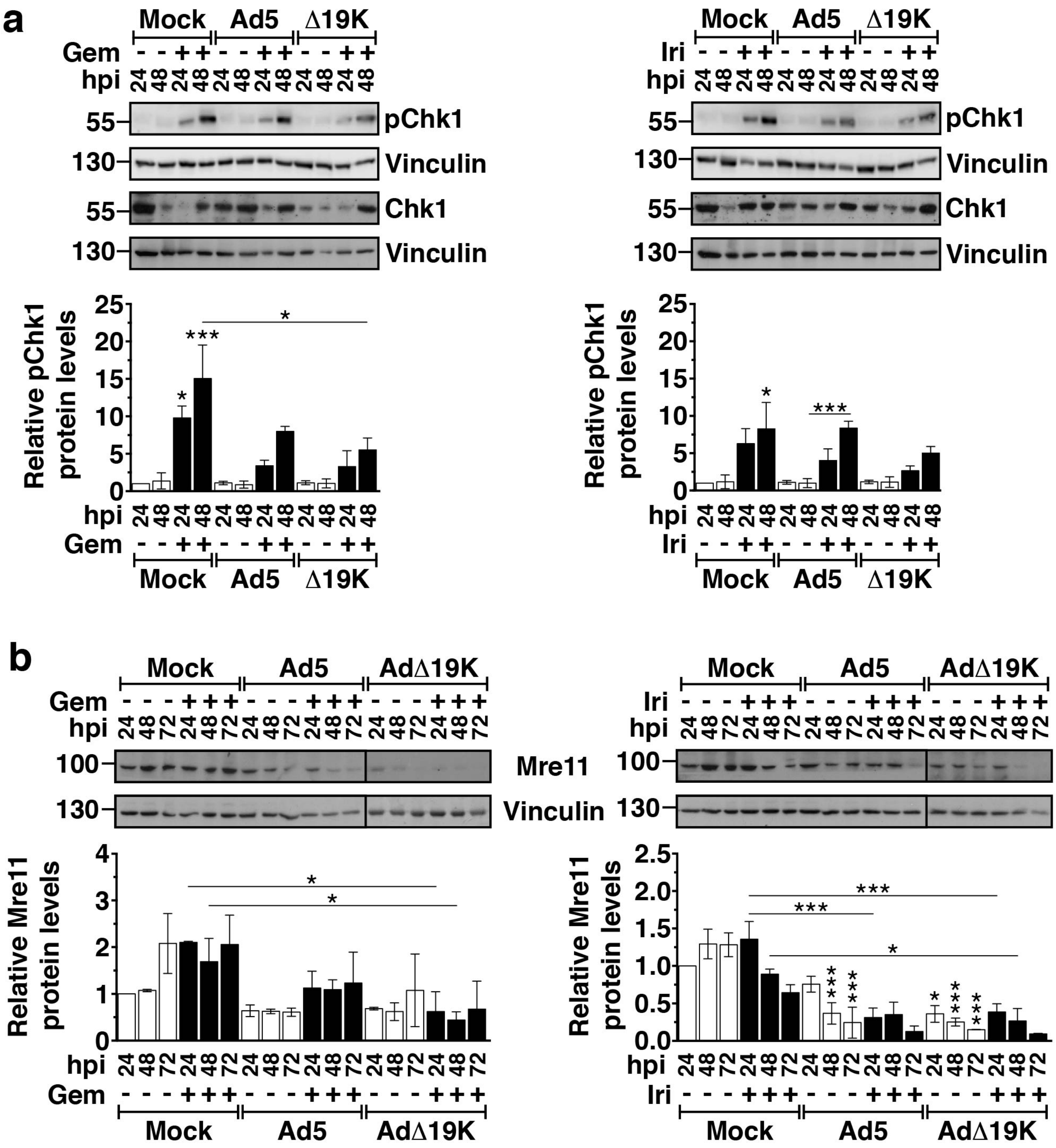


**Supplementary Figure S4: AdΔ19K attenuates the DNA damage response induced by gemcitabine and irinotecan in MIAPaCa-2 cells. a-b.** Immunoblot analysis of phospho-Chk1 (Ser296) (pChk1) and total Chk1 A. and Mre11 B. in MIAPaCa-2 cells, treated with 300ppc viruses ± 20nM gemcitabine (Gem) **(left panels)** and 15µM irinotecan (Iri) **(right panels)**. Numbers indicate MW size marker (kDa). **a. Upper panels:** Representative immunoblots of phospho- and total Chk1 (56kDa) with Vinculin (130kDa) as loading control. **Bottom panels:** pChk1 protein levels were quantified by densitometric analysis, normalised to total Chk1 and the loading control and expressed as fold-change relative to mock 24h (=1). Error bars represent S.E.M. of 2 independent experiments. **b. Upper panels:** Representative immunoblots of Mre11 (81kDa) with Vinculin (130kDa) as loading control. **Bottom panels:** Mre11 protein levels were quantified by densitometric analysis, normalised to the loading control and expressed as fold-change relative to mock 24h (=1). Error bars represent S.E.M. of at least 3 independent experiments. **a-b.** *.p<0.05 **.p<0.01, ***.p<0.001 (one-way ANOVA with Bonferroni's multiple comparison test).

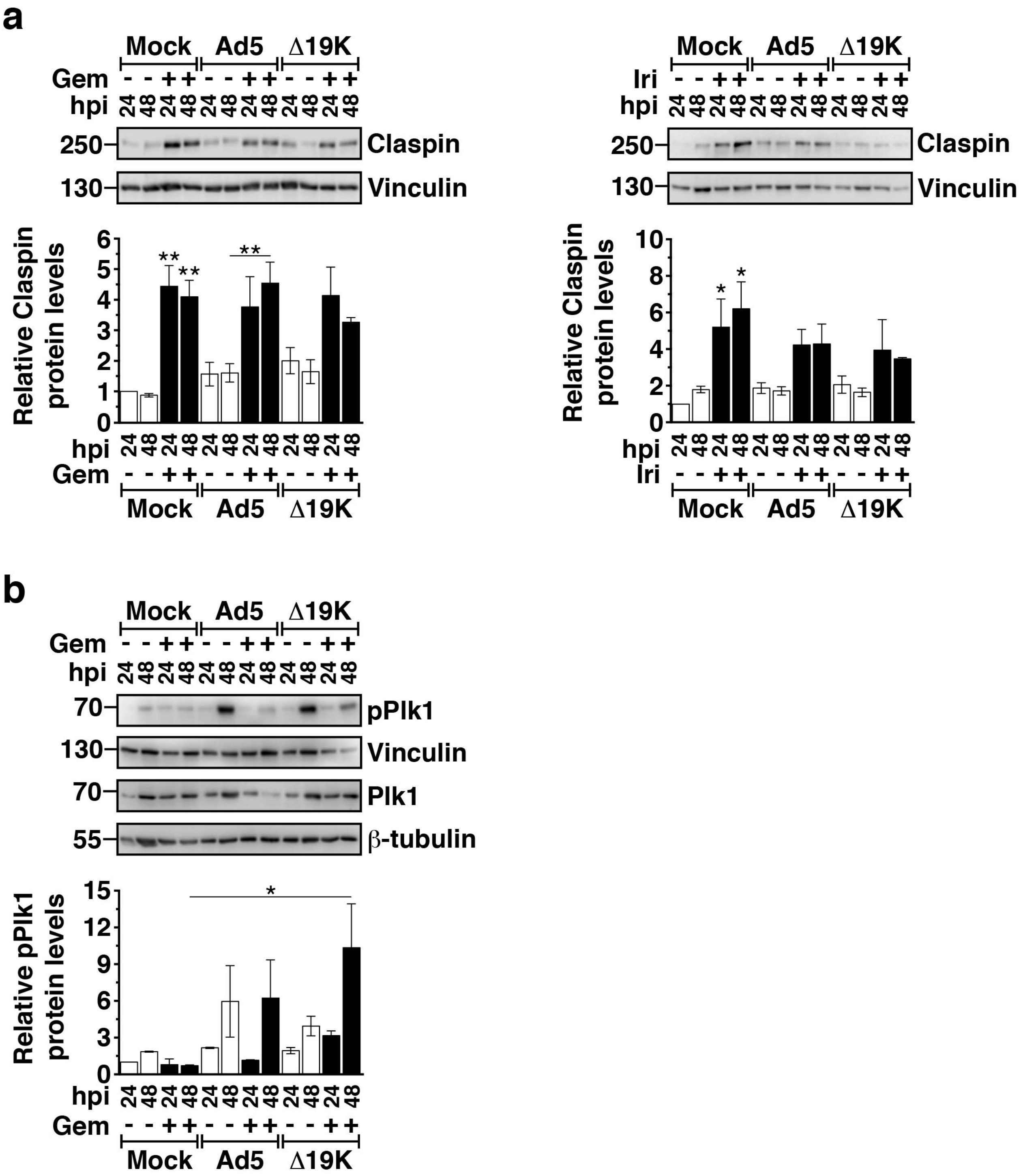


**Supplementary Figure S5: Claspin and phospho-Plk1 expression in MIAPaCa-2 cells. a.** Immunoblot analysis of Claspin in MIAPaCa-2 cells, treated with 300ppc viruses ± 20nM gemcitabine (Gem) **(left panel)** or 15μM irinotecan (Iri) **(right panel)**. **Upper panels:** Representative immunoblots of Claspin (250kDa) with Vinculin (130kDa) as loading control. Numbers indicate MW size marker (kDa). **Bottom panels:** Claspin protein levels were quantified by densitometric analysis, normalised to the loading control and expressed as fold-change relative to mock 24h (=1). Error bars represent S.E.M. of 3 independent experiments. **b.** Immunoblot analysis of phospho-Plk1 (T210) (pPlk1) and total Plk1 in MIAPaCa-2 cells, treated with 300ppc viruses ± 20nM gemcitabine (Gem). **Upper panel:** Representative immunoblots of phospho- and total Plk1 (68kDa) with Vinculin (130kDa) or β-Tubulin (55kDa) as loading controls. Numbers indicate MW size marker (kDa). **Bottom panel:** pPlk1 protein levels were quantified by densitometric analysis, normalised to total Plk1 and the loading control and expressed as fold-change relative to mock 24h (=1). Error bars represent S.E.M. of 2 independent experiments. **a-b.** *.p<0.05, **.p<0.01 (one-way ANOVA with Bonferroni's multiple comparison test).

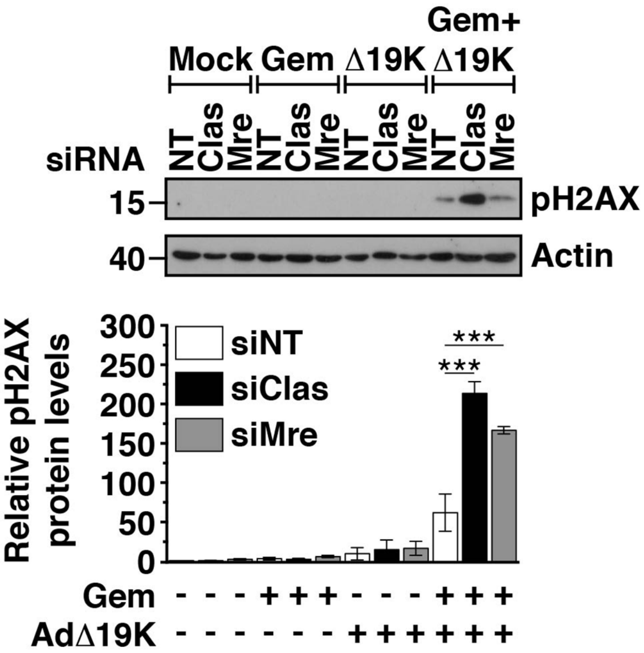


**Supplementary Figure S6: Claspin and Mre11 knockdown enhance DNA damage induced by AdΔ19K and gemcitabine in PT45 cells.** PT45 cells were transfected with siRNA against Claspin (siClas), Mre11 (siMre) or non-targeting siRNA (siNT) and treated with 300ppc viruses ± 5nM gemcitabine (Gem). At 72hpi cells were prepared for immunoblot analysis of phospho-histone H2AX (Ser139) (pH2AX). **Upper panel:** Representative immunoblot of pH2AX (15kDa) with Actin (42kDa) as loading control. Numbers indicate MW size marker (kDa). **Bottom panel:** pH2AX protein levels were quantified by densitometric analysis, normalised to the loading control and expressed as fold-change relative to mock 24h (=1). Error bars represent S.E.M. of 3 independent experiments. ***.p<0.001 (one-way ANOVA with Bonferroni's multiple comparison test).

**Supplementary Table 1: Theoretical and experimentally obtained additive values ± S.E.M.**

| | | | Gem + Ad5 | Gem + AdΔ19K | Iri + Ad5 | Iri + AdΔ19K |
|---|---|---|---|---|---|---|
| **PT45** | **48h** | Theoretical | 6.0±1.9 | 7.5±2.0 | 7.0±2.4 | 7.8±3.9 |
| | | Experimental | 5.9±0.6 | 9.5±2.7 | 8.2±2.3 | 11.2±1.3 |
| | **72h** | Theoretical | 17.8±5.0 | 19.9±5.0 | 13.7±3.4 | 17.5±5.0 |
| | | Experimental | 20.3±2.7 | 41.3±6.7* | 20.3±5.8 | 39.7±5.4 |
| | **96h** | Theoretical | 47.4±11.2 | 50.4±13.1 | 60.0±5.7 | 67.0±4.4 |
| | | Experimental | 46.5±2.1 | 67.0±4.1* | 43.0±4.5 | 75.0±1.8 |
| **MIAPaCa-2** | **48h** | Theoretical | 9.3±5.1 | 8.4±2.7 | 14.0±5.4 | 12.8±3.2 |
| | | Experimental | 8.8±3.7 | 10.3±2.4 | 9.8±4.9 | 13.6±5.7 |
| | **72h** | Theoretical | 32.5±10.5 | 29.6±2.7 | 46.3±15.9 | 42.3±4.7 |
| | | Experimental | 25.0±6.7 | 36.0±4.3 | 36.0±8.0 | 42.0±3.4 |
| | **96h** | Theoretical | 77.0±14.0 | 84.9±9.1 | 101.0±22.8 | 106.0±9.5 |
| | | Experimental | 54.0±5.3 | 60.9±5.0 | 64.8±1.4 | 79.5±4.5 |

[*] $p<0.05$ (one-way ANOVA with Bonferroni's Multiple Comparison Test)

**Supplementary Table 2: Statistical significance for G1- and G2-phase presented in Figure 2A**

| | | G1 | G2 | | | G1 | G2 |
|---|---|---|---|---|---|---|---|
| **24h** | Mock vs Ad5 | ns | ns | **60h** | Mock vs Ad5 | *** | ** |
| | Mock vs Δ19K | ns | ns | | Mock vs Δ19K | *** | *** |
| | Mock vs Gem | * | ns | | Mock vs Gem | ** | ns |
| | Ad5 vs Δ19K | ns | ns | | Ad5 vs Δ19K | ns | ns |
| | Ad5 vs Gem+Ad5 | ns | ns | | Ad5 vs Gem+Ad5 | *** | ns |
| | Δ19K vs Gem+Δ19K | * | ns | | Δ19K vs Gem+Δ19K | ns | ns |
| | Gem vs Gem+Ad5 | ns | ns | | Gem vs Gem+Ad5 | *** | *** |
| | Gem vs Gem+Δ19K | ns | ns | | Gem vs Gem+Δ19K | *** | ** |
| | Gem+Ad5 vs Gem+Δ19K | ns | ns | | Gem+Ad5 vs Gem+Δ19K | ns | ns |
| **36h** | Mock vs Ad5 | ** | ns | **72h** | Mock vs Ad5 | *** | *** |
| | Mock vs Δ19K | ** | ns | | Mock vs Δ19K | *** | ** |
| | Mock vs Gem | *** | ns | | Mock vs Gem | ns | ns |
| | Ad5 vs Δ19K | ns | ns | | Ad5 vs Δ19K | ns | ns |
| | Ad5 vs Gem+Ad5 | ns | ns | | Ad5 vs Gem+Ad5 | *** | * |
| | Δ19K vs Gem+Δ19K | ns | ns | | Δ19K vs Gem+Δ19K | *** | ns |
| | Gem vs Gem+Ad5 | ns | ns | | Gem vs Gem+Ad5 | *** | *** |
| | Gem vs Gem+Δ19K | ns | * | | Gem vs Gem+Δ19K | *** | *** |
| | Gem+Ad5 vs Gem+Δ19K | ns | ns | | Gem+Ad5 vs Gem+Δ19K | ns | ns |
| **48h** | Mock vs Ad5 | *** | *** | | | | |
| | Mock vs Δ19K | *** | *** | | | | |
| | Mock vs Gem | *** | * | | | | |
| | Ad5 vs Δ19K | ns | ns | | | | |
| | Ad5 vs Gem+Ad5 | ns | ns | | | | |
| | Δ19K vs Gem+Δ19K | ns | ns | | | | |
| | Gem vs Gem+Ad5 | ** | ns | | | | |
| | Gem vs Gem+Δ19K | *** | ns | | | | |
| | Gem+Ad5 vs Gem+Δ19K | ns | ns | | | | |

* $p<0.05$, ** $p<0.01$, *** $p<0.001$, ns non-significant. (one-way ANOVA with Bonferroni's Multiple Comparison Test)
